# Supplementary material for: Nuclear lactate dehydrogenase A senses ROS to produce α-hydroxybutyrate for HPV-induced cervical tumor growth
Source: Nat Commun. 2018 Oct 24;9:4429. doi: 10.1038/s41467-018-06841-7 (PMC6200739; doi:10.1038/s41467-018-06841-7)

Supplementary information for

Nuclear Lactate Dehydrogenase A Senses ROS to Produce α-Hydroxybutyrate for HPV-Induced Cervical Tumor Growth

Yuan Liu, *et al*.

Supplementary Figure 1

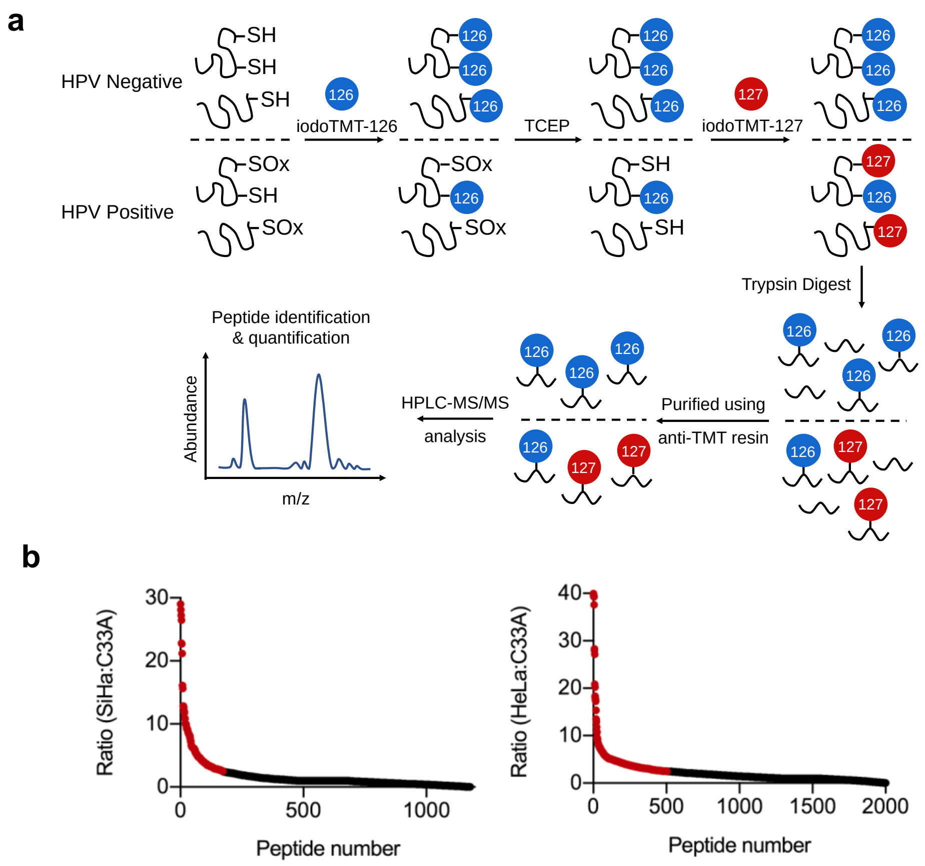

Supplementary Figure 1. Mass spectrometry screening potential redox sensors responding to HPV infection. **a** Schematic of iodoTMT-based redox proteomics approach. HPLC-MS/MS, high performance liquid chromatography - tandem mass spectrometry. **b** 173 peptides (SiHa vs C33A) and 505 peptides (HeLa vs C33A) were identified to be oxidized in response to HPV. Peptides IodoTMT 127/126 ratios for cysteine-containing peptides in SiHa or HeLa versus C33A cells. Red data points mark the ratios ≥ 2.5, which was used as a cutoff for HPV-dependent changes in cysteine reactivity.

Supplementary Figure 2

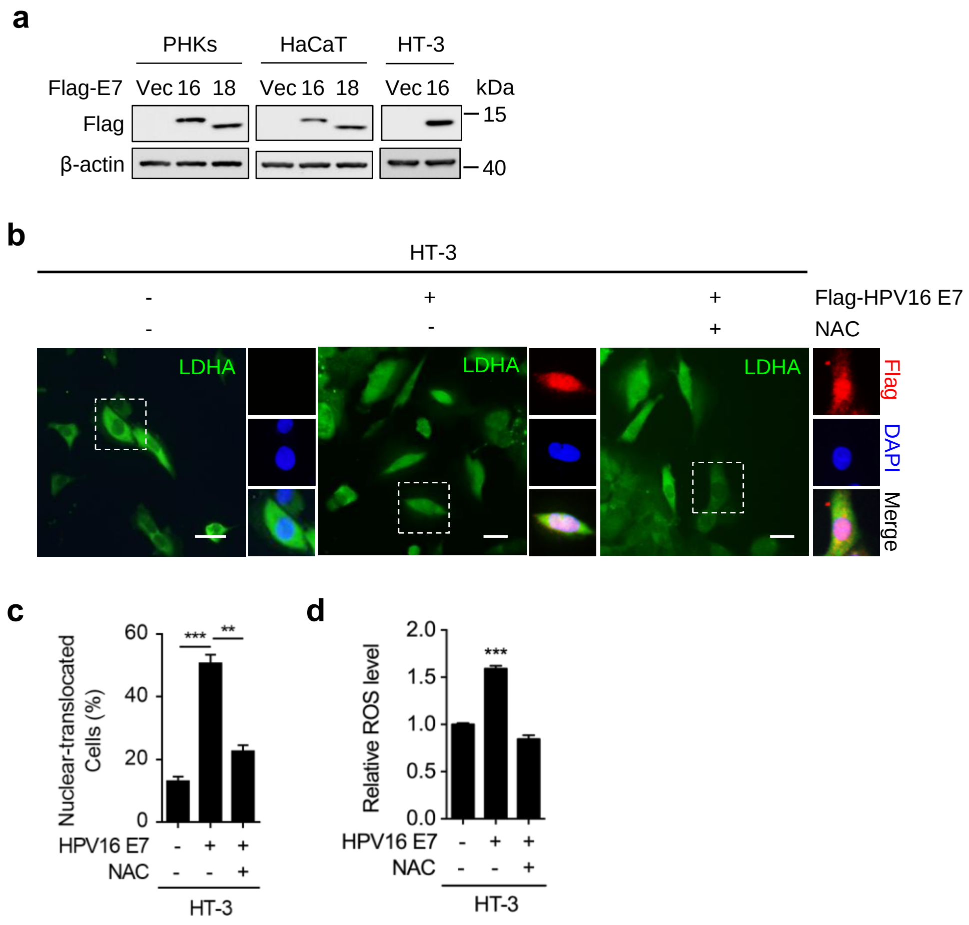

Supplementary Figure 2. HPV16 E7 promotes LDHA nuclear translocation in a ROS-dependent manner. **a** Generation of primary human cervix keratinocytes (PHKs), HaCaT, and HT-3 cells expressing Flag-tagged HPV16/18 E7 using retrovirus system. Flag-HPV16/18 E7 expression level was determined by western blotting. **b, c** HPV16 E7 promotes LDHA nuclear translocation in a ROS-dependent manner. HT-3 cells stably expressing vector or Flag-tagged HPV16 E7 were treated with or without 1 mM NAC for 6 hr, followed by staining with anti-LDHA (green), anti-Flag (red) antibodies and DAPI (blue). Scale bars, 10 μm (**b**). The percentage of cells with nucleus-localized LDHA compared to total cell number was quantified (**c**). **d** HPV16 E7 enhances ROS production. Cellular ROS were measured in HT-3 cells stably expressing vector or HPV16 E7 coupled with or without 1 mM NAC treatment for 6 hr, followed by using the ROS-sensitive fluorescent dye (CM-H_2DCFDA) with flow

cytometry according to the manufacturer's protocol. Results shown are representative of 3 independent experiments. All data are shown as mean ± s.e.m. The p values were determined by two-tailed t-test. The values of $p<0.05$ were considered statistically significant. *, **, and *** denote $p<0.05$, $p<0.01$, and $p<0.001$, respectively. NS means non significant.

Supplementary Figure 3

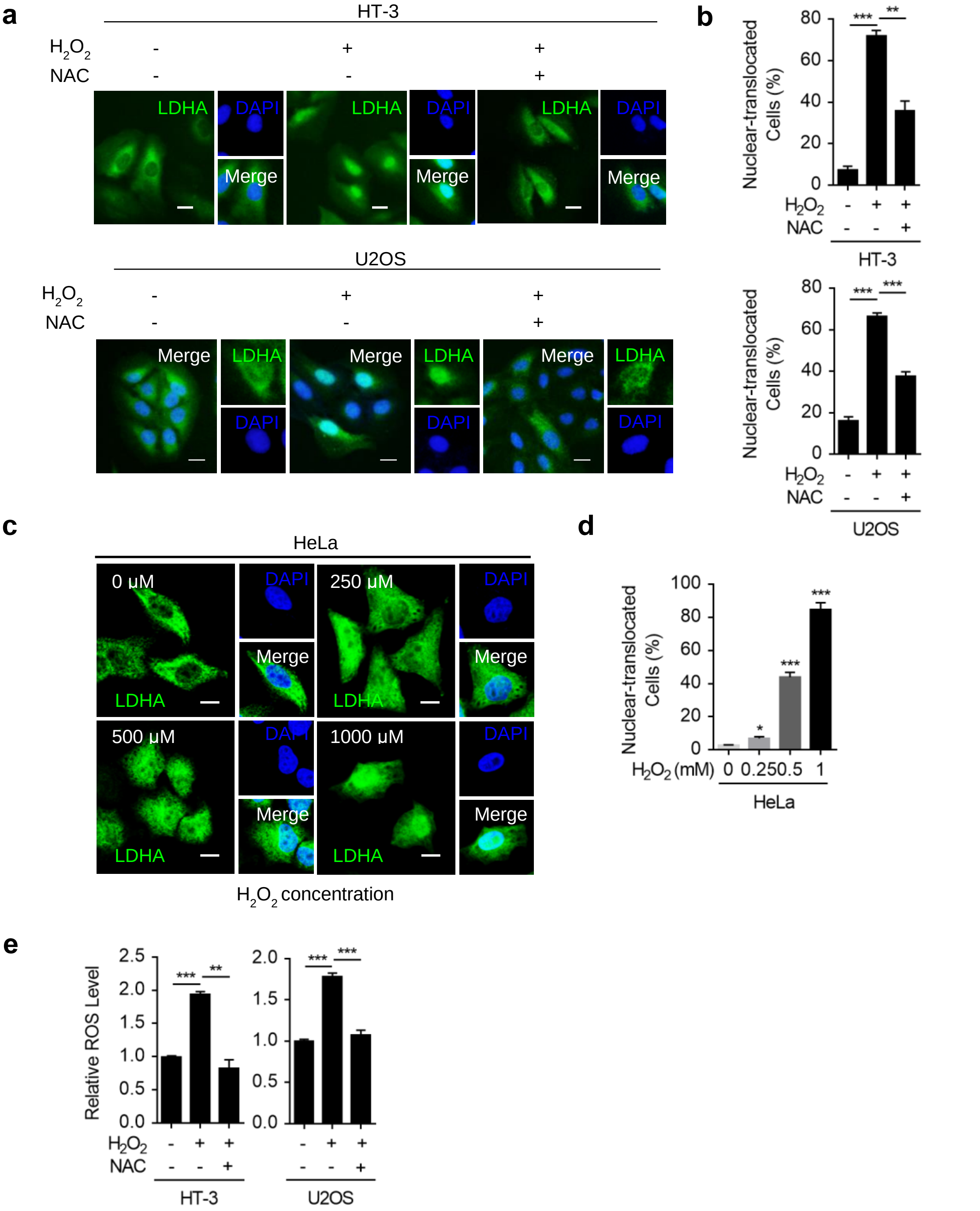

Supplementary Figure 3. ROS promote LDHA nuclear translocation. **a, b** ROS promote LDHA nuclear translocation. Immunofluorescent images of LDHA (green) in HT-3 and U2OS cells upon 10 μM H_2O_2 treatment for 6 hr, with or without 1 mM NAC supplement for extended 6 hr as indicated. DAPI, blue. Scale bars, 10 μm (**a**). The percentage of cells with nucleus-localized LDHA compared to total cell number was quantified (**b**). **c, d** LDHA nuclear translocation is profoundly increased in an

H_2O_2-dose dependent manner. Immunofluorescent images of LDHA (green) in HeLa cells upon different dose of H_2O_2 treatment as indicated. DAPI, blue. Scale bars, 10 μm (**c**). The percentage of cells with nucleus-localized LDHA normalized against the total cell number was quantified (**d**). **e** H_2O_2 treatment enhances ROS production. Cellular ROS were measured in HT-3 and U2OS cells upon 10 μM H_2O_2 treatment for 6 hr, with or without 1 mM NAC supplement for extended 6 hr as indicated, followed by using the ROS-sensitive fluorescent dye (CM-H_2DCFDA) with flow cytometry according to the manufacturer's protocol. Results shown are representative of 3 independent experiments. All data are shown as mean ± s.e.m. The *p* values were determined by two-tailed *t*-test. The values of $p<0.05$ were considered statistically significant. *, **, and *** denote $p<0.05$, $p<0.01$, and $p<0.001$, respectively. NS means non significant.

Supplementary Figure 4

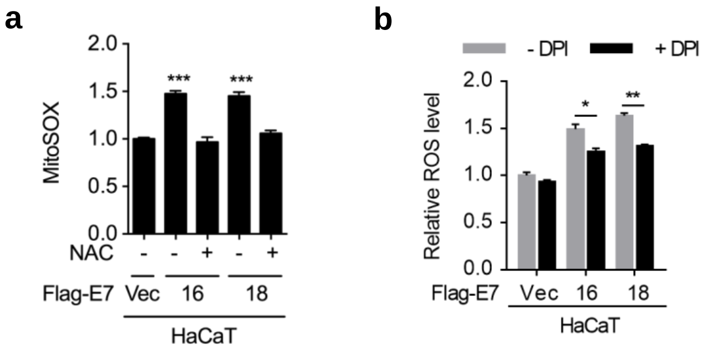

Supplementary Figure 4. Both mitochondrial and NOXs contribute to HPV16/18 E7-induced intracellular ROS elevation. **a** HPV16/18 E7 upregulates mitochondrial ROS production. Mitochondrial ROS were measured in HaCaT cells stably expressing vector or Flag-tagged HPV16/18 E7 were treated with or without 1 mM NAC for 6 hr, followed by using the mitochondrial superoxide indicator (MitoSOX) with flow cytometry according to the manufacturer's protocol. **b** DPI reduces the cellular ROS level responding to HPV16/18 E7. Cellular ROS were measured in HaCaT cells stably expressing vector or Flag-tagged HPV16/18 E7 were treated with or without 10 μM Diphenyleneiodonium chloride (DPI) for 24 hr as indicated, followed by using the ROS-sensitive fluorescent dye (CM-H_2DCFDA) with flow cytometry according to the manufacturer's protocol. Results shown are representative of 3 independent experiments. All data are shown as mean ± s.e.m. The *p* values were determined by two-tailed *t*-test. The values of $p<0.05$ were considered statistically significant. *, **, and *** denote $p<0.05$, $p<0.01$, and $p<0.001$, respectively. NS means non significant.

Supplementary Figure 5

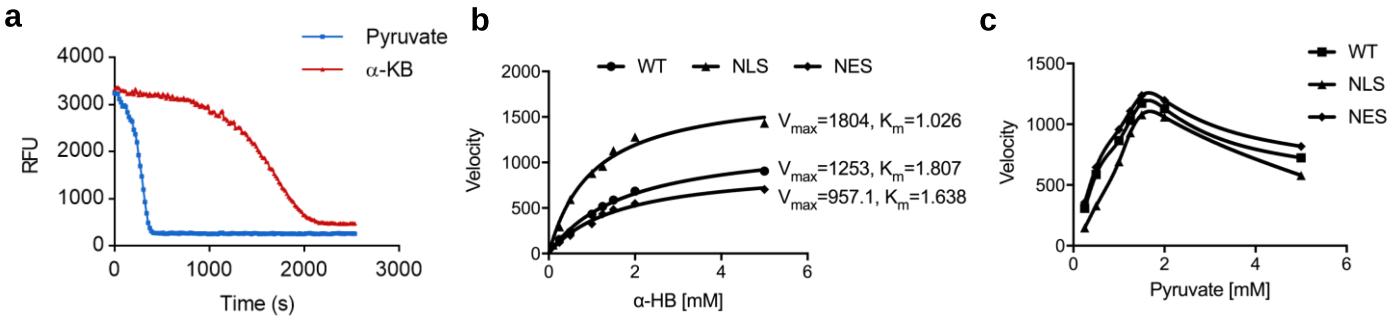

Supplementary Figure 5. Nuclear LDHA gains a non-canonical enzyme activity to produce α-HB. **a** Enzyme assay measuring NADH consumption by LDHA immunoprecipitated using anti-Flag beads from HEK293T transfected with Flag-LDHA^{NLS} plasmid in reactions with 2 mM pyruvate or 2 mM α-KB. **b, c** LDHA^{NLS} presents a higher V_{max} and a lower K_m value of the non-canonical enzyme activity, but not the canonical activity. Michaelis-Menten kinetics analysis of LDHA canonical and non-canonical enzyme activities using different substrate, α-KB (**b**) and pyruvate (**c**), concentrations. WT, wildtype; NLS, nuclear localization signal; NES, nuclear export signal. Results shown are representative of 3 independent experiments.

Supplementary Figure 6

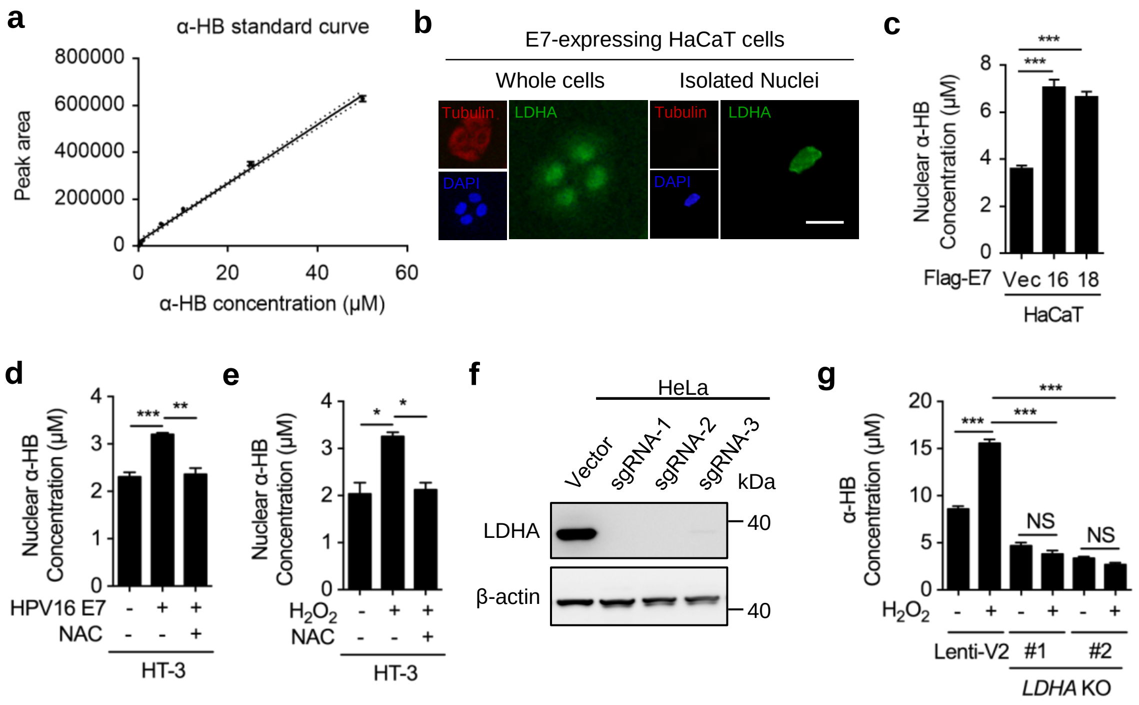

Supplementary Figure 6. HPV16/18 E7 expression or ROS stimulation accumulates nuclear α-HB. **a** Standard curve of α-HB concentration determined by LC-MS/MS. The standard curve was determined by using the linear regression which was drawn on the ratio of the peak area of the seven internal standards measured by LC-MS/MS. **b** Identification of nuclear isolation protocol. The whole cells and the isolated nuclei of HaCaT cells stably expressing HPV16 E7 were immunostained with anti-LDHA (green), anti-Tubulin (red) antibodies and DAPI (blue). Scale bars, 10 μm. **c-e** HPV16/18 E7 expression or ROS stimulation accumulates nuclear α-HB. The extracted metabolite samples from the isolated nucleus of HaCaT cells stably expressing vector or HPV16/18 E7 (**c**) and the isolated nucleus of HT-3 cells stably expressing vector or HPV16 E7 coupled with or without 1 mM NAC treatment for 6 hr (**d**) and HT-3 cells treated with or without 10 μM H_2O_2 for 6 hr, and supplemented with or without 1mM NAC for extended 6 hr (**e**) as indicated were analyzed by LC-MS/MS. **f** Generation of HeLa *LDHA* KO cells. LentiCRISPRv2 system was used to knockout *LDHA* in HeLa cells though three different sgRNAs. *LDHA* KO efficiencies were determined by western blotting. **g** Accumulated α-HB is produced

by LDHA. The extracted metabolite samples from vector or *LDHA* knockout (KO) HeLa cells treated with or without 10 μM H_2O_2 for 6 hr as indicated were analyzed by LC-MS/MS. Results shown are representative of 3 independent experiments. All data are shown as mean ± s.e.m. The *p* values were determined by two-tailed *t*-test. The values of $p<0.05$ were considered statistically significant. *, **, and *** denote $p<0.05$, $p<0.01$, and $p<0.001$, respectively. NS means non significant.

Supplementary Figure 7

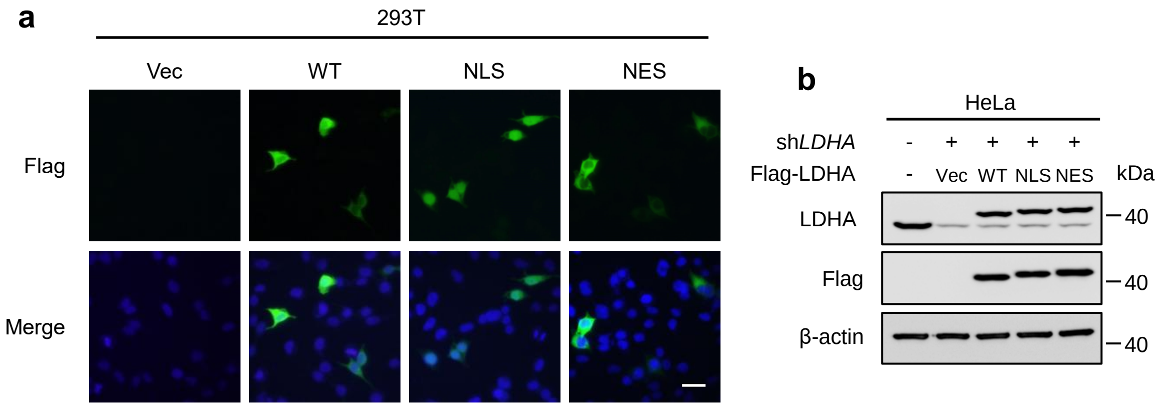

Supplementary Figure 7. Generation of stable HeLa cells with *LDHA* knockdown and LDHA Vec/WT/NLS/NES rescue. **a** LDHA^{NLS} and LDHA^{NES} are localized in the nucleus and cytoplasm, respectively. Immunofluorescent images of Flag (green) in HEK293T cells transient transfected with vector (Vec), Flag-tagged wildtype (WT), nuclear localization signal (NLS), and nuclear export signal (NES) LDHA plasmids as indicated. DAPI, blue. Scale bars, 10 µm. **b** Generation of stable HeLa cells with *LDHA* knockdown and rescue. shRNA-resistant LDHA^{WT}, LDHA^{NLS}, or LDHA^{NES} mutant was re-introduced into HeLa cells with stable knockdown of endogenous *LDHA*. *LDHA* knockdown efficiency and re-expression were determined by western blotting. Results shown are representative of 3 independent experiments.

Supplementary Figure 8

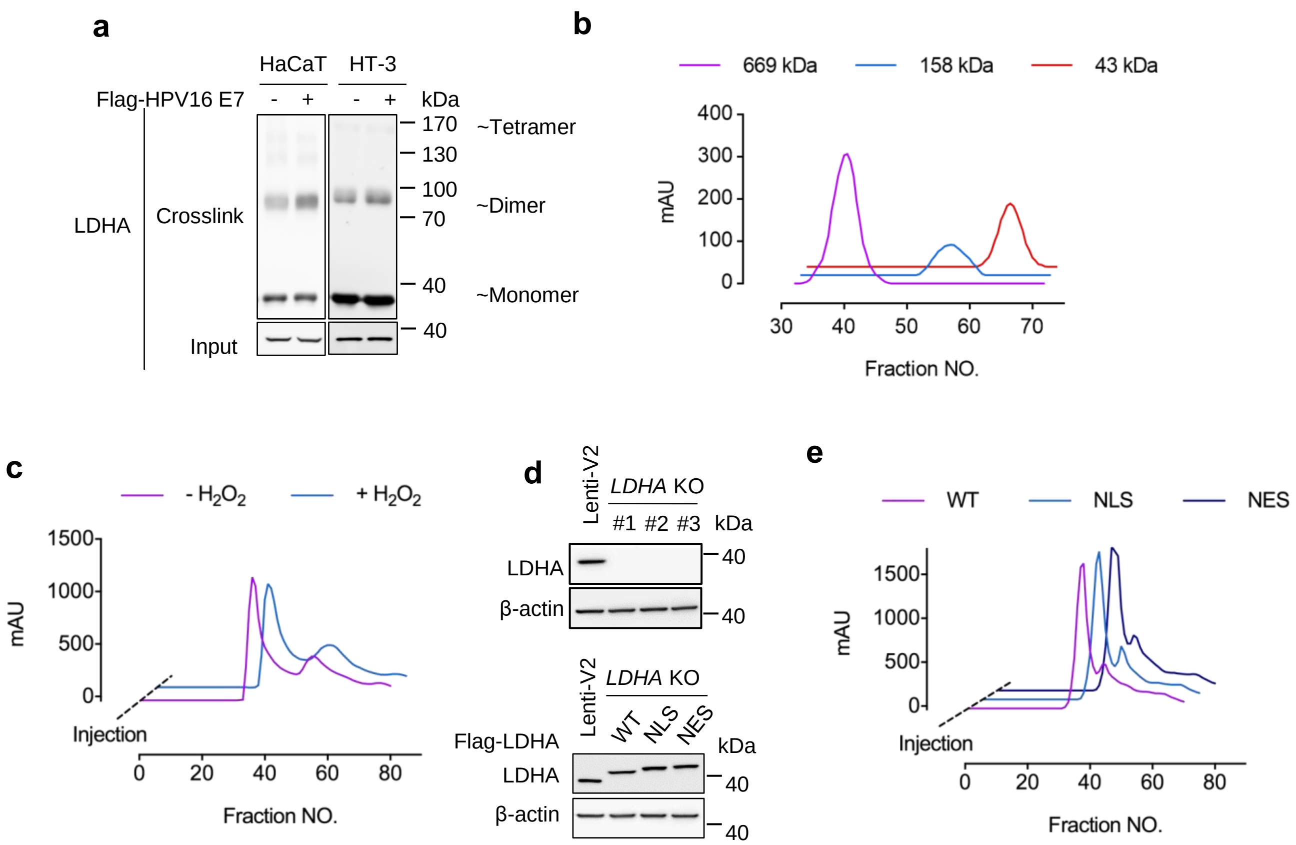

Supplementary Figure 8. ROS disrupt LDHA tetramer formation and promote non-canonical enzyme activity. **a** HPV16 E7 expression increases LDHA dimer formation. Cell lysate of HaCaT and HT-3 cells stably expressing vector or HPV16 E7 were crosslinked by 0.025 % glutaraldehyde and analyzed by western blotting using LDHA antibody. **b** Gel filtration molecular mass was determined by Thyroglobulin (669 kDa), Aldolase (158 kDa), and Ovalbumin (43 kDa). **c** Gel filtration of HT-3 whole cell lysate with or without H_2O_2 treatment. Extracts were passed over the gel filtration column. The speed rate of flow is 0.4 ml/min. Fractions were collected every 0.25 ml per tube. Purple (- H_2O_2) and blue (+ H_2O_2) curves stood for absorbance (mAU) at 280 nm. **d** Generation of HEK293T *LDHA* KO cells with LDHA$^{WT/NLS/NES}$ rescue. LentiCRISPRv2 system was used to knockout HEK293T *LDHA* though three different sgRNAs. LDHA^{WT}, LDHA^{NLS}, or LDHA^{NES} mutant was re-introduced using retrovirus overexpression systems. *LDHA* KO and WT/NLS/NES LDHA rescue efficiencies were determined by western blotting. **e** Gel filtration of

HEK293T *LDHA* KO cells with LDHA$^{WT/NLS/NES}$ rescue. Extracts were passed over the gel filtration column. The speed rate of flow is 0.4 ml/min. Fractions were collected every 0.25 ml per tube. Purple (WT), blue (NLS), and dark blue (NES) curves stood for absorbance (mAU) at 280 nm. Results shown are representative of 3 independent experiments.

Supplementary Figure 9

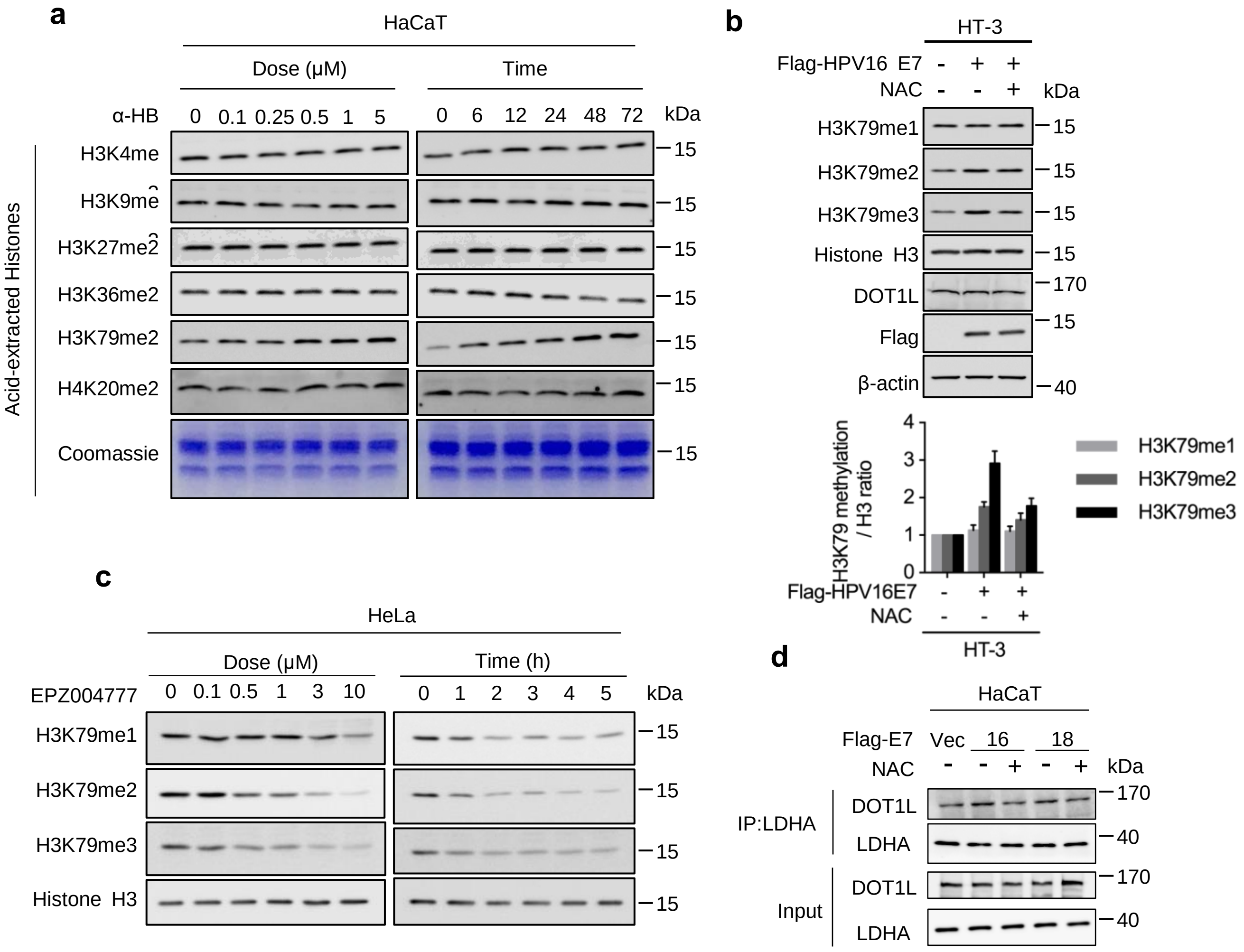

Supplementary Figure 9. DOT1L mediates α-HB-induced H3K79 hypermethylation. **a** Histone H3K79 di-methylation levels are upregulated by α-HB in a dosage and time-dependent manner. HaCaT cells were treated with or without different dose and time of sodium α-HB as indicated for western blotting using site-specific methylation antibodies. Acid-extracted histones were visualized by Commassie staining, as loading control. **b** HPV16 E7 expression enhances H3K79 tri-methylation. H3K79 methylation levels and DOT1L expression were analyzed in HT-3 cells stably expressing vector or Flag-tagged HPV16 E7 coupled with or without 1 mM NAC treatment for 6 hr. **c** DOT1L methyl-transferase activity is inhibited by EPZ004777 in a dosage and time-dependent manner. HeLa cells were treated with or without different dose and time of EPZ004777 as indicated for western blotting using specific

methylation antibodies. Histone H3 used as loading control. **d** HPV16/18 E7 triggers the binding of DOT1L with LDHA. Endogenous co-immunoprecipitation of LDHA in HaCaT cells stably expressing vector or HPV16/18 E7 coupled with or without 1 mM NAC treatment for 6 hr, and blotting of LDHA and DOT1L. Results shown are representative of 3 independent experiments.

Supplementary Figure 10

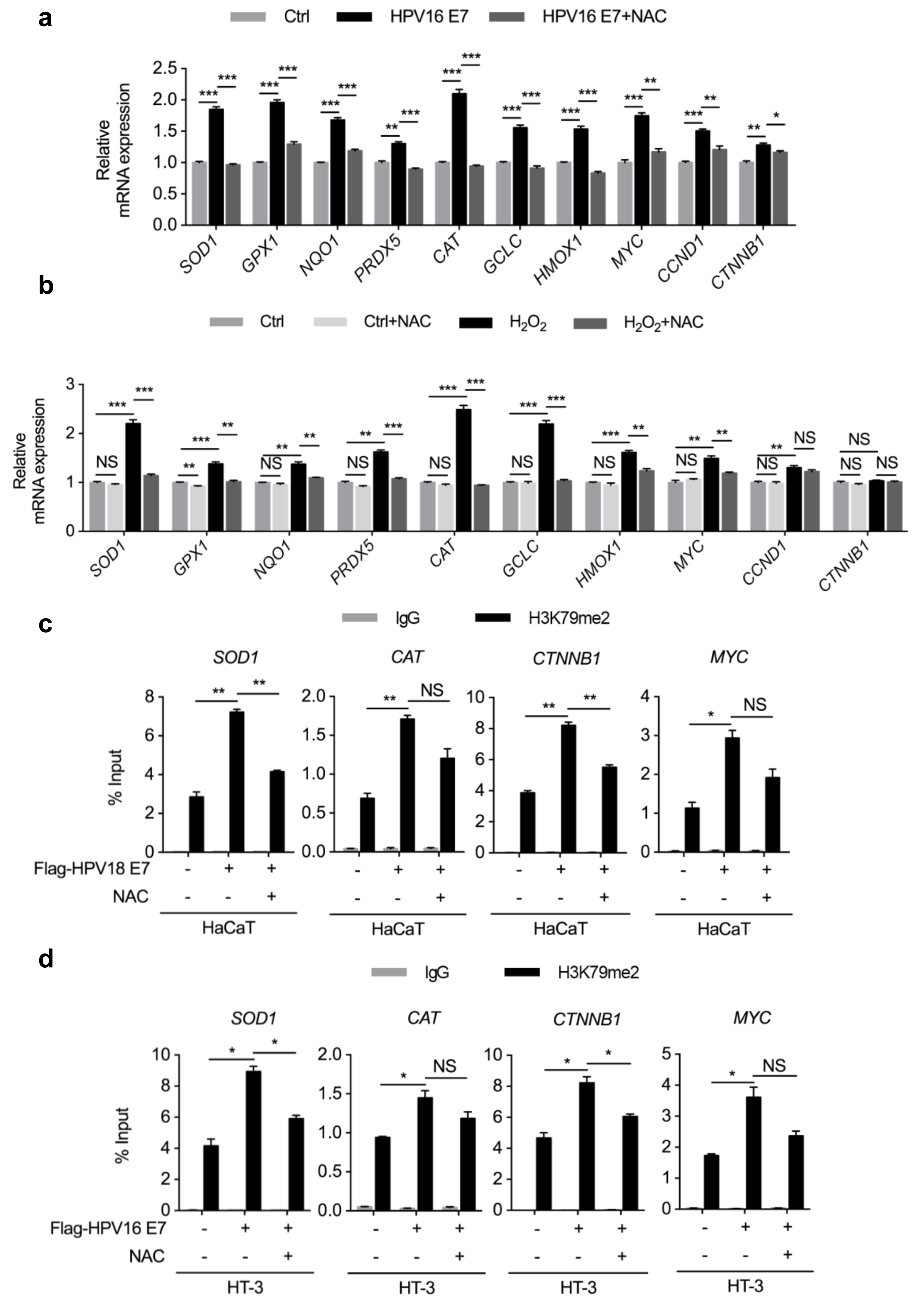

Supplementary Figure 10. E7 induces the expression of antioxidant and Wnt target genes though accumulation of H3K79 di-methylation level. **a, b,** HPV16 E7 or ROS induce the expression of antioxidant and Wnt target genes. qPCR detecting

antioxidant and Wnt target genes in HT-3 cells stably expressing vector or HPV16 E7 coupled with or without 1mM NAC treatment for 6 hr (**a**), or treated with or without 10 μM H_2O_2 for 6 hr and/or 1mM NAC for 6 hr (**b**) as indicated. **c, d** HPV16/18 E7 accumulates the H3K79 di-methylation level at *SOD1*, *CAT*, *CTNNB1*, and *MYC* gene body. ChIP-qRT-PCR showing the percentage of H3K79me2 enrichment at *SOD1*, *CAT*, *CTNNB1*, and *MYC* gene body relative to input genomic DNA in HaCaT cells stably expressing vector or Flag-tagged HPV18 E7 treated with or without 1mM NAC for 6 hr (**c**) and HT-3 cells stably expressing vector or Flag-tagged HPV16 E7 treated with or without 1mM NAC for 6 hr (**d**). Rabbit IgG was included as a negative control. Results shown are representative of 3 independent experiments. All data are shown as mean ± s.e.m. The *p* values were determined by two-tailed *t*-test. The values of $p<0.05$ were considered statistically significant. *, **, and *** denote $p<0.05$, $p<0.01$, and $p<0.001$, respectively. NS means non significant.

Supplementary Figure 11

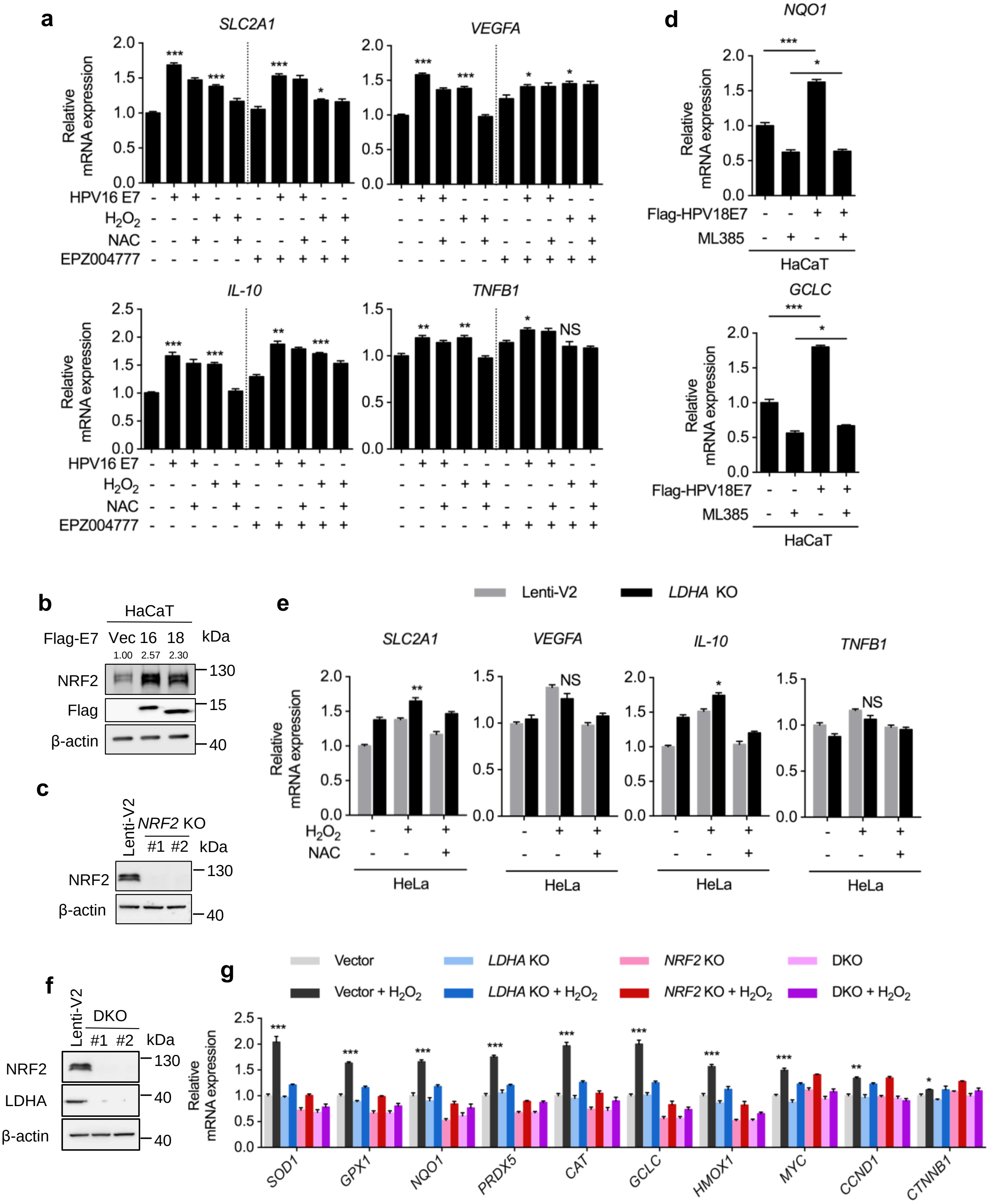

Supplementary Figure 11. NRF2 is required for nuclear LDHA-induced antioxidant responses. **a** EPZ004777 fails to block increased *SLC2A1*, *VEGFA*, *TNFB1*, and *IL-10*

gene expression induced by E7 and H_2O_2. qPCR detecting *SLC2A1*, *VEGFA*, *IL-10,* and *TNFB1* genes in vector (treated with or without 10 μM H_2O_2 for 6 hr) or HPV16 E7 in HaCaT cells coupled with or without 1 mM NAC for 6 hr and 3 μM EPZ004777 for 24 hr as indicated. **b** E7 upregulates NRF2 protein level. Cell extract from vector or HPV16/18 E7-expressing HaCaT cells were analyzed by western blotting. β-actin used as loading control. **c** Generation of HeLa *NRF2* KO cells. LentiCRISPRv2 system was used to knockout *NRF2* through two different sgRNAs in HeLa cells. *NRF2* KO efficiencies were determined by western blotting. **d** NRF2 inhibition blocks increased *NQO1* and *GCLC* gene expression induced by E7. qPCR detecting *NQO1* and *GCLC* genes in vector or HPV18 E7-expressing HaCaT cells treated with or without 10 μM ML385 for 24 hr (**f**) as indicated. **e** *LDHA* KO fails to decrease *SLC2A1*, *VEGFA*, *TNFB1*, and *IL-10* gene expression activated by H_2O_2. qPCR detecting *SLC2A1*, *VEGFA*, *TNFB1*, and *IL-10* genes in HeLa *LDHA* KO cells upon 10 μM H_2O_2 for 6 hr with or without 1 mM NAC treatment for extended 6 hr as indicated. **f** Generation of HeLa *LDHA* and *NRF2* double KO (DKO) cells. LentiCRISPRv2 system was used to knockout *LDHA* and *NRF2* though two different sgRNAs in HeLa cells, respectively. DKO efficiencies were determined by western blotting. **g** *LDHA* KO, or *LDHA NRF2* DKO block increased gene expression upon H_2O_2 treatment, while *NRF2* KO block the activation of antioxidant genes but not Wnt target genes. qPCR detecting antioxidant and Wnt target genes in HeLa *LDHA* KO, *NRF2* KO or *LDHA NRF2* DKO cells treated with or without 10 μM H_2O_2 for 6 hr. Results shown are representative of 3 independent experiments. All data are shown as mean ± s.e.m. The p values were determined by two-tailed t-test. The values of $p<0.05$ were considered statistically significant. *, **, and *** denote $p<0.05$, $p<0.01$, and $p<0.001$, respectively. NS means non significant.

Supplementary Figure 12

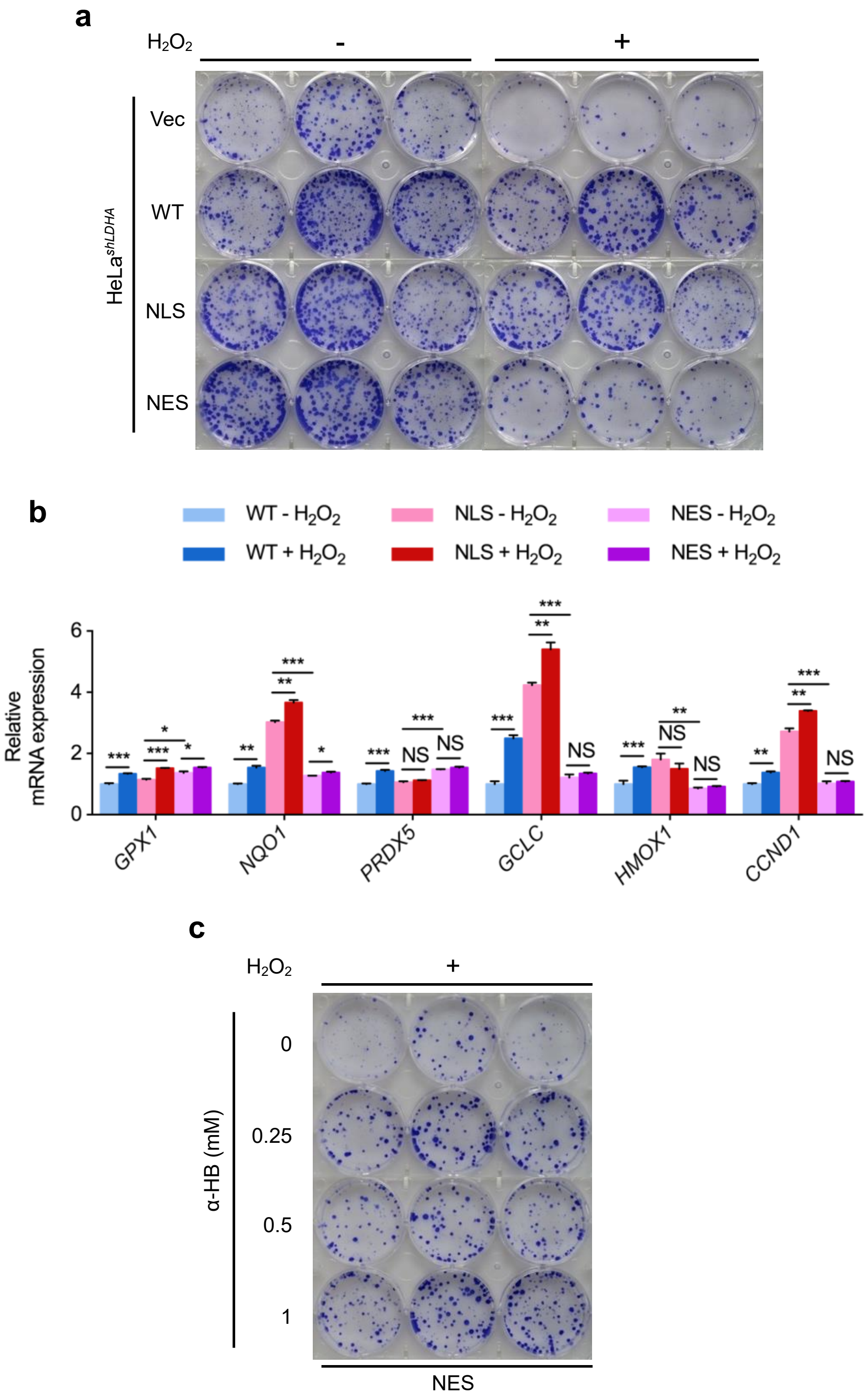

Supplementary Figure 12. Nuclear LDHA produced α-HB protects cervical cancer cells from oxidative stress. **a** Nuclear LDHA increases colony formation under oxidative stress. Representative wells of the colony formation experiment depicted in Fig. 6c. **b** ROS enhance target gene expressions in LDHA^{WT} and LDHA^{NLS} but not

LDHA^{NES} stable cells. qPCR of antioxidant and Wnt target genes in HeLa stable cells with *LDHA* knockdown and Vec/WT/NLS/NES rescue with or without 10 μM H_2O_2 treatment for 6 hr. **c** α-HB restores LDHA^{NES} colony formation under oxidative stress. Representative wells of the colony formation experiment depicted in Fig. 6i. Results shown are representative of 3 independent experiments. All data are shown as mean ± s.e.m. The *p* values were determined by two-tailed *t*-test. The values of $p<0.05$ were considered statistically significant. *, **, and *** denote $p<0.05$, $p<0.01$, and $p<0.001$, respectively. NS means non significant.

Supplementary Figure 13

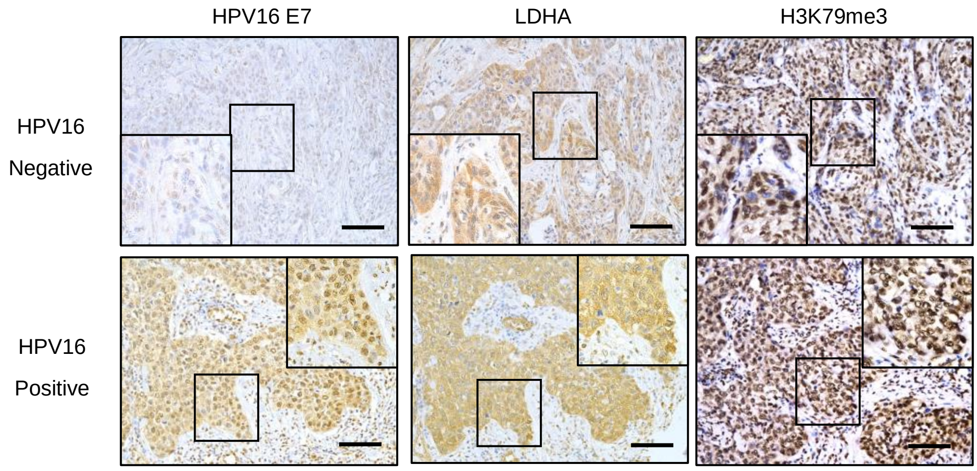

Supplementary Figure 13. HPV16 E7-induced LDHA nuclear translocation implicates in cervical cancer development. HPV16 E7 level, LDHA nuclear translocation and H3K79 tri-methylation are positively correlated with each other in human cervical tumor tissues. Representative IHC images of HPV16E7 expression, LDHA localization, and H3K79me3 level in HPV16 negative and positive cervical tumor samples. Scale bar, 100 μm.

Supplementary Figure 14. Uncropped blots and gels.

Figure 1h

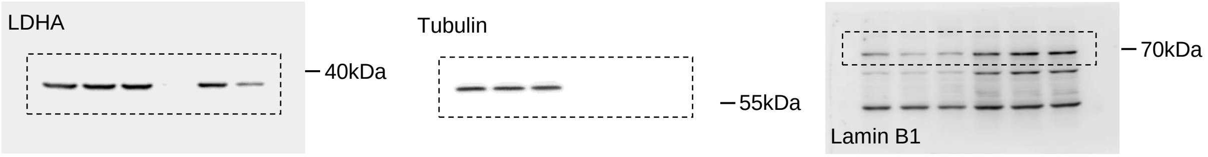

Figure 3a

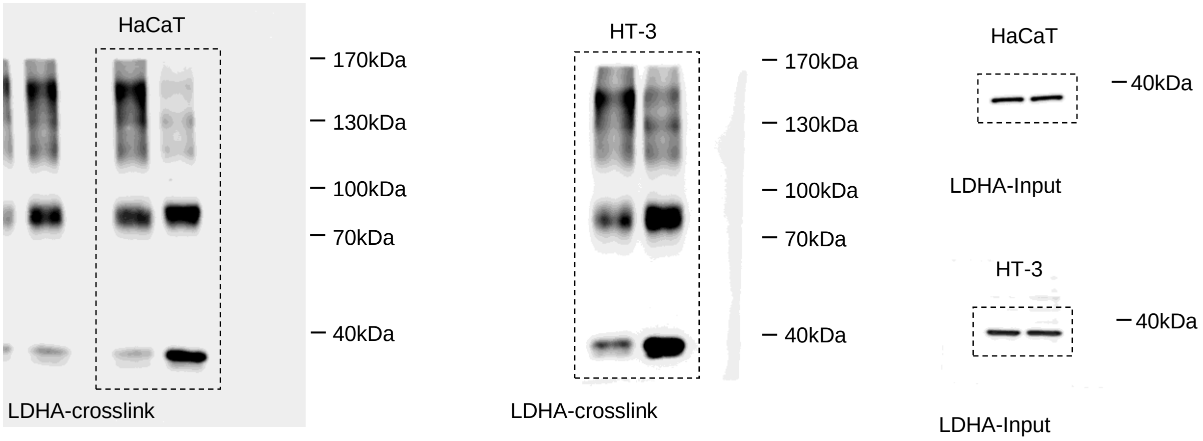

Figure 3b

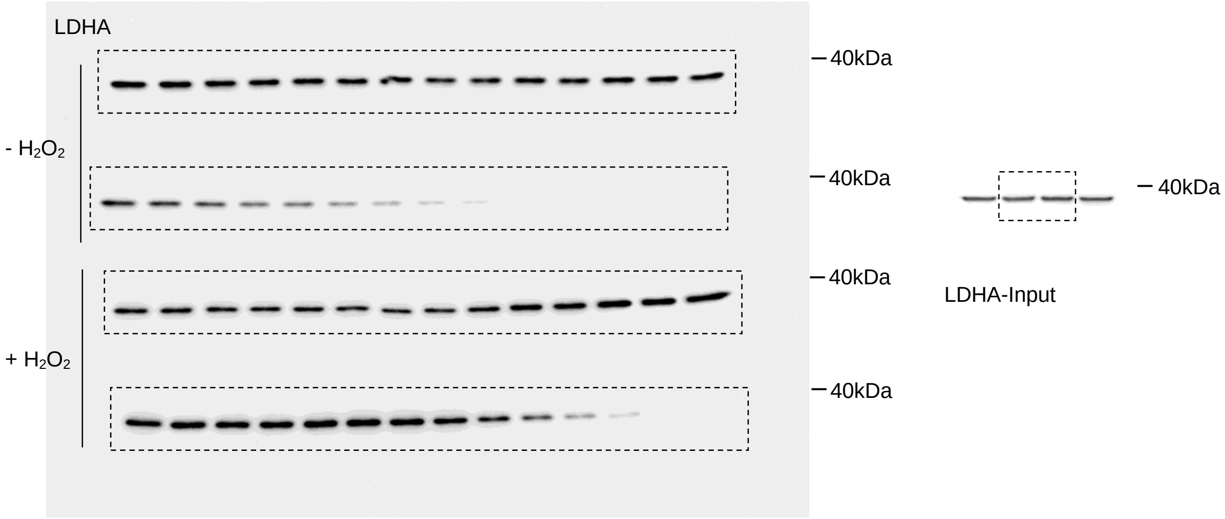

Figure 3d

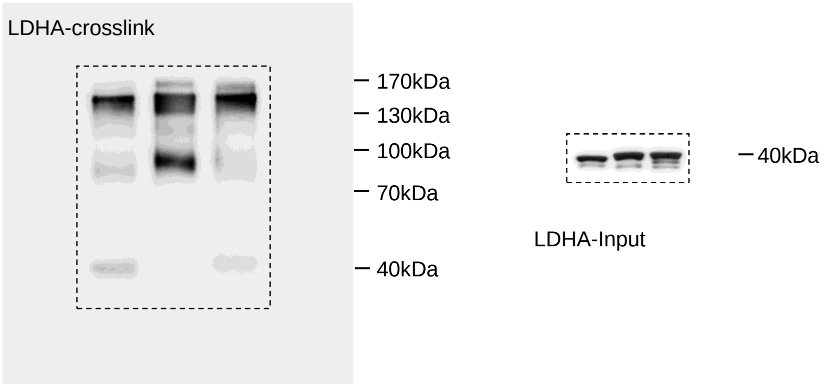

Figure 3e

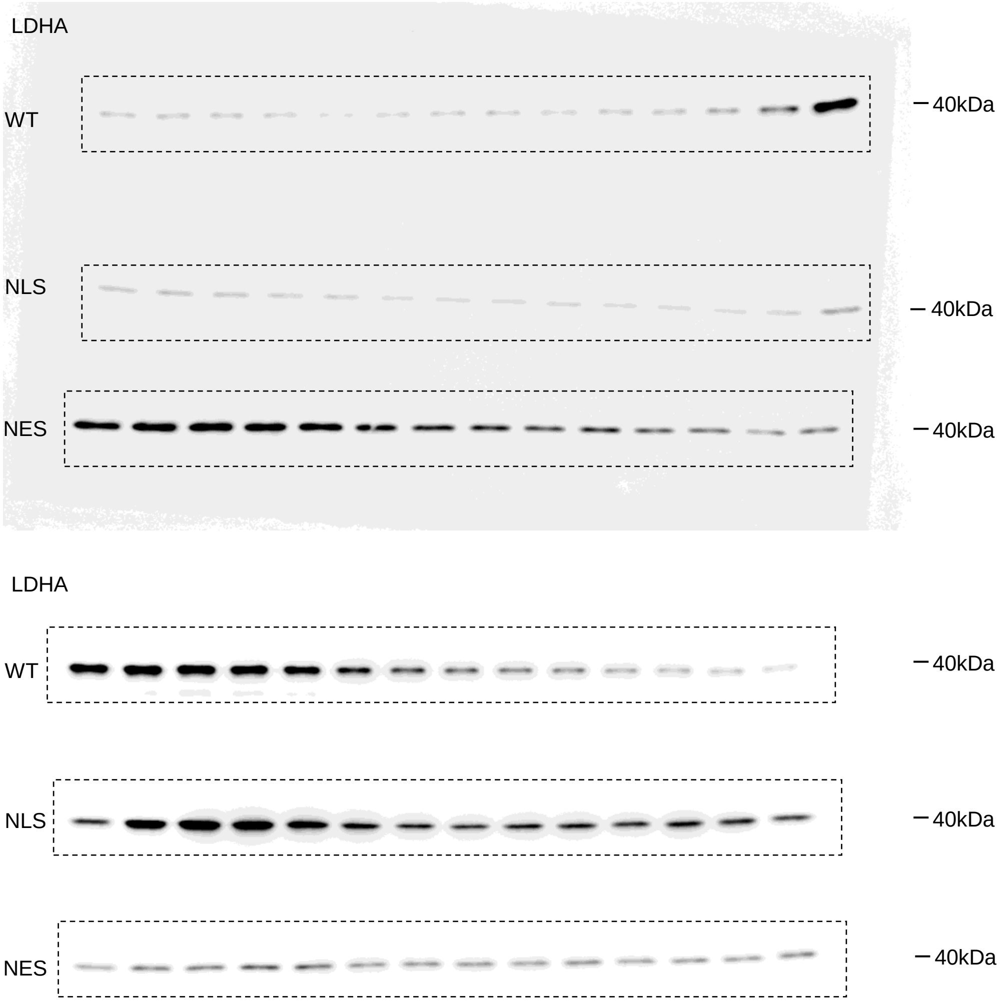

Figure 4a

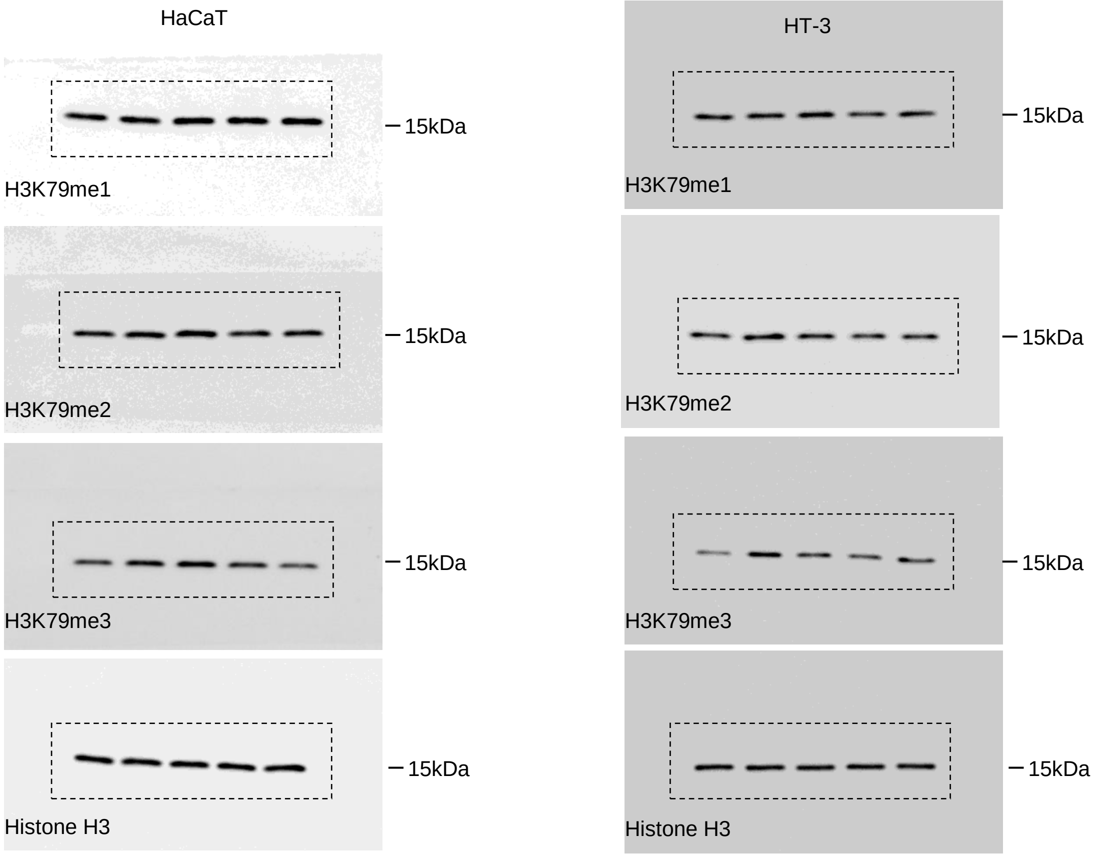

Figure 4b

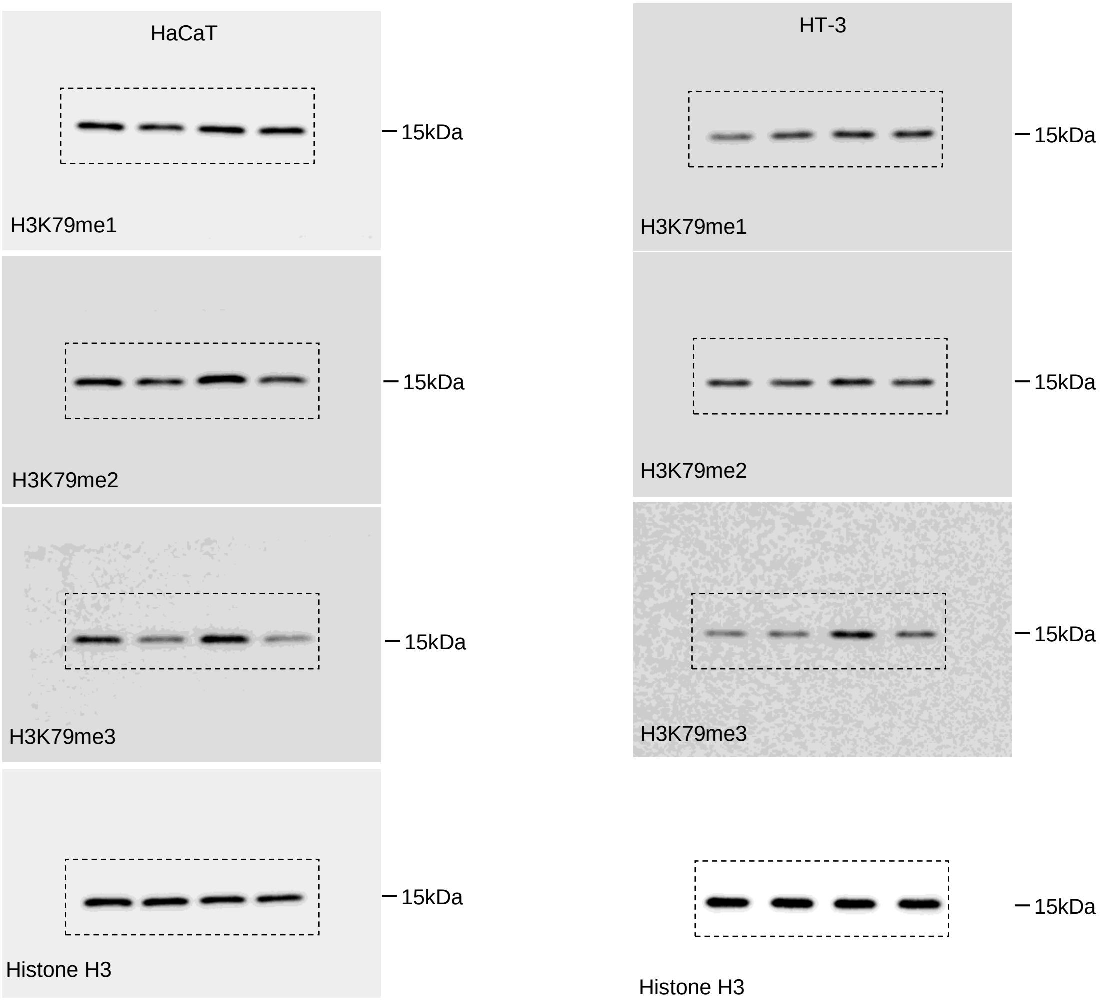

Figure 4c

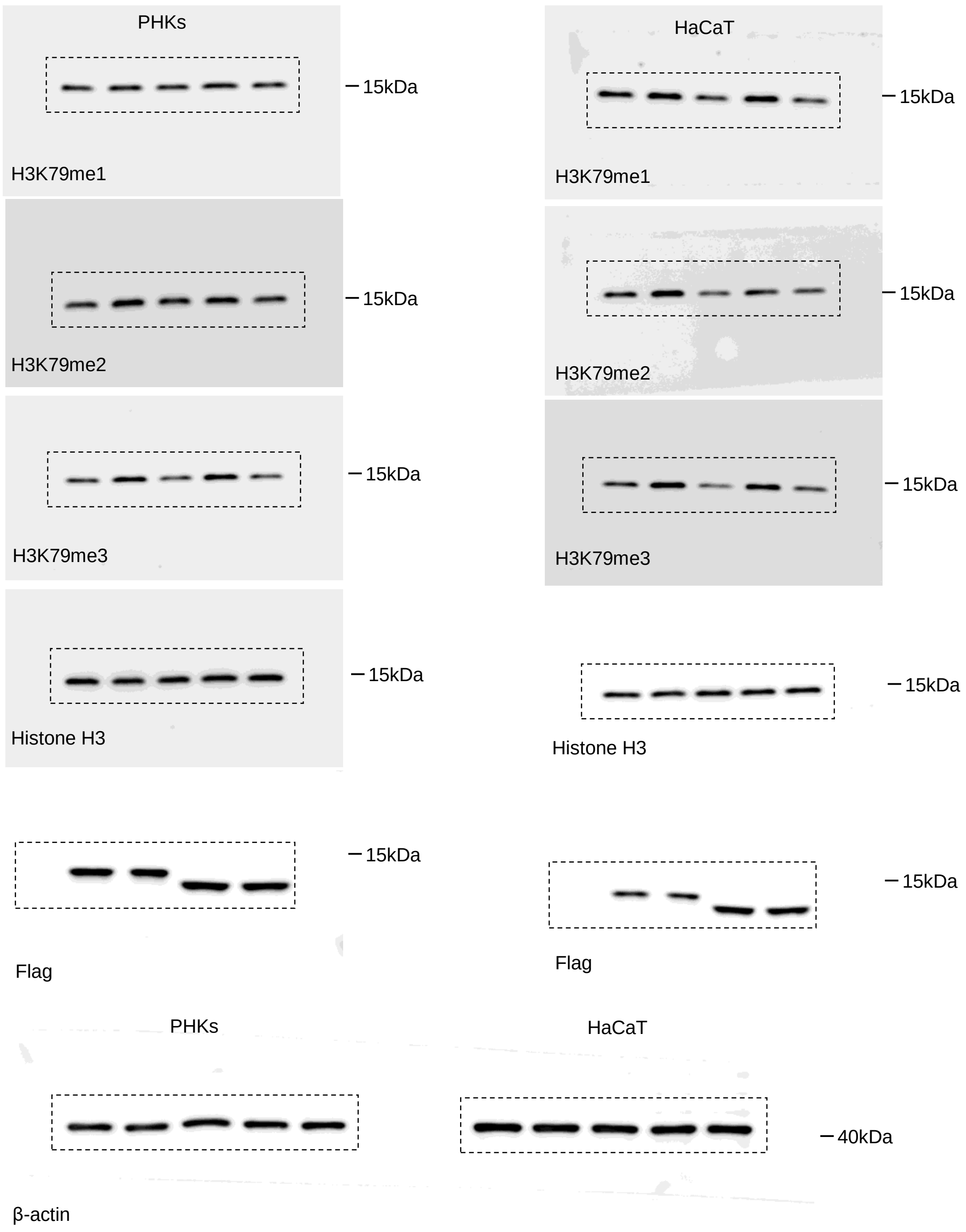

Figure 4d

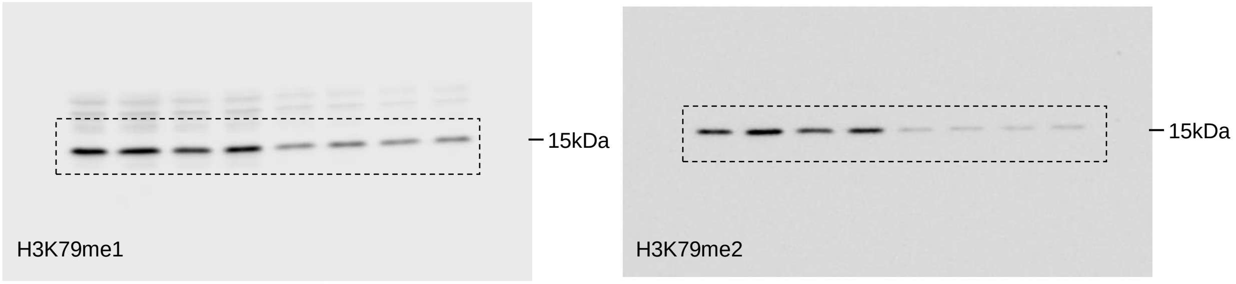

Figure 4d

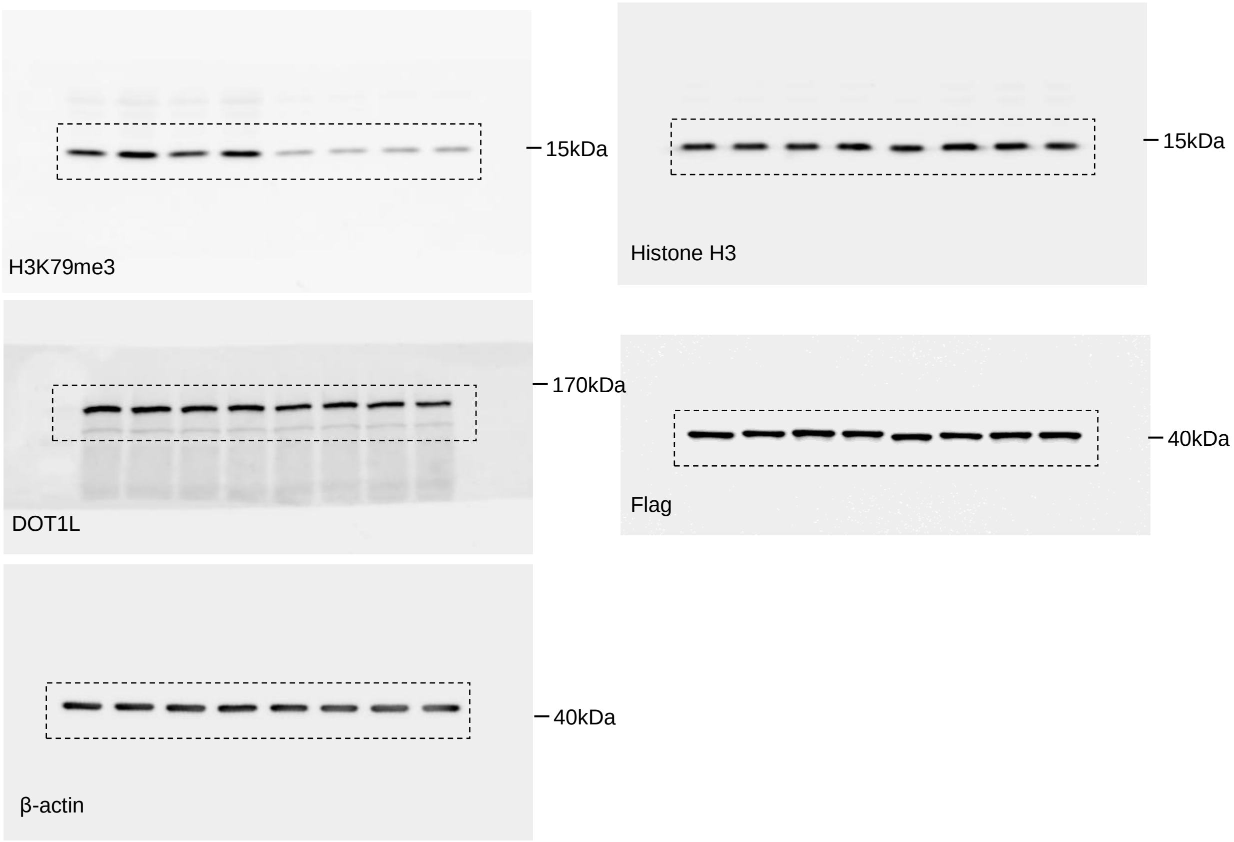

Figure 4e

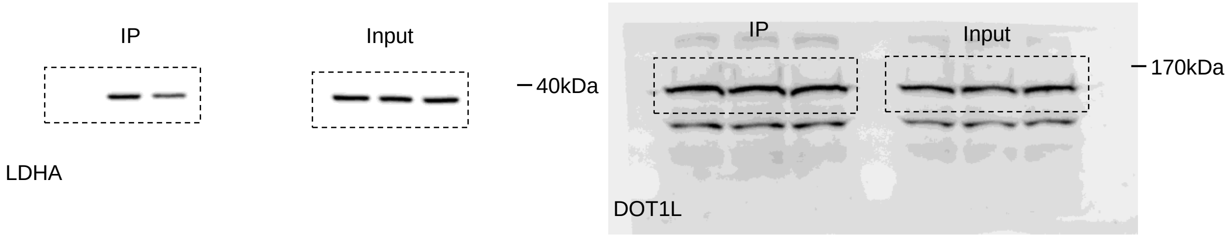

Figure 4f

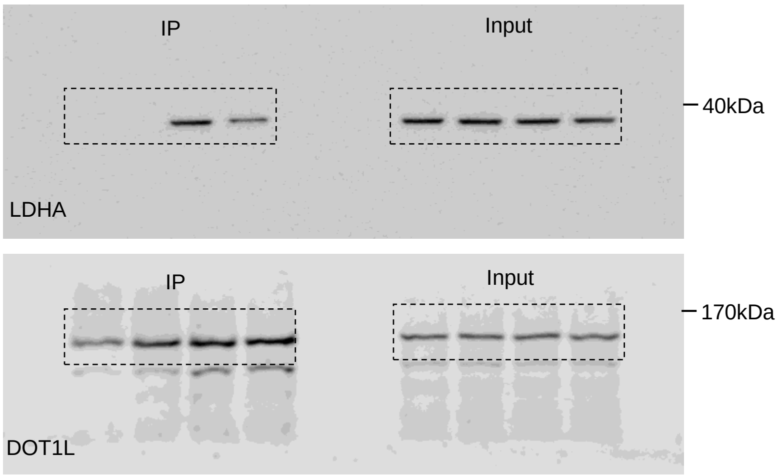

Figure 4g

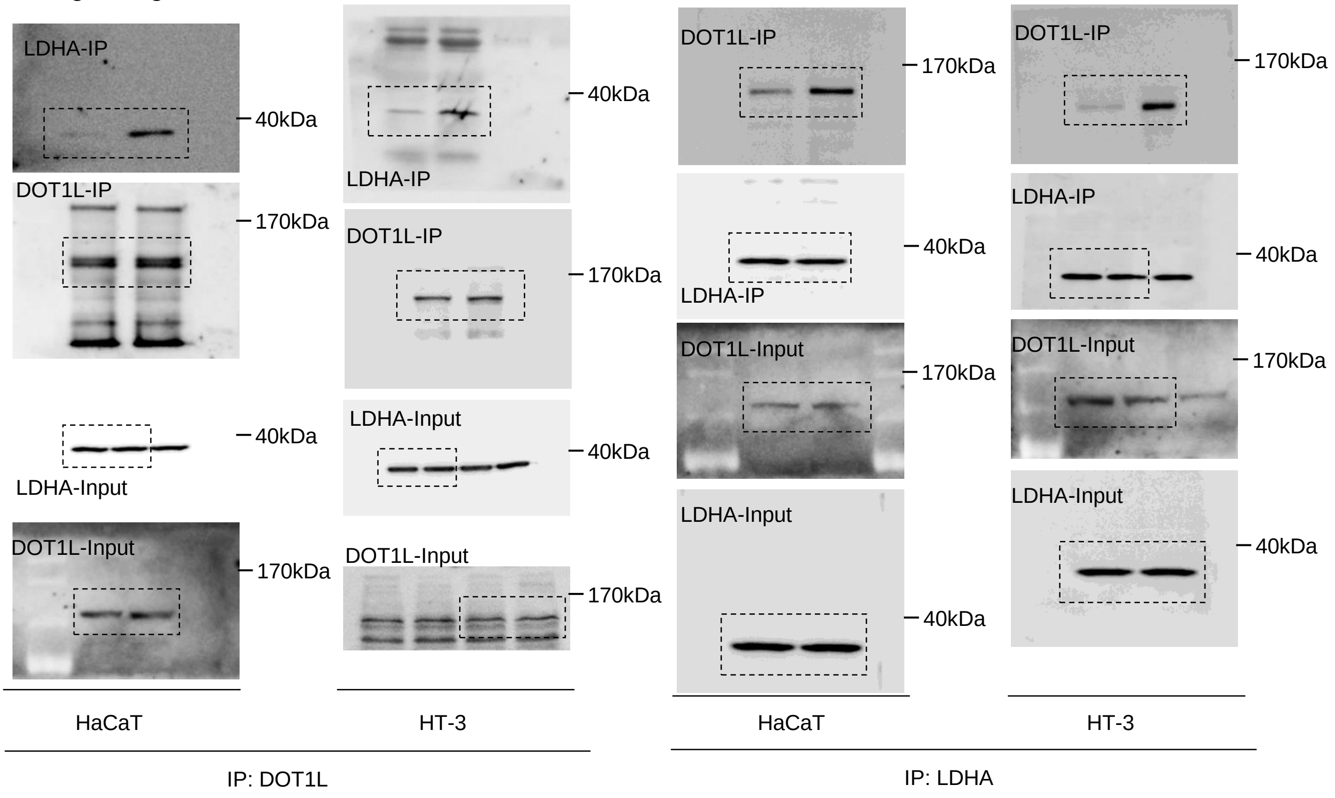

Figure 7d

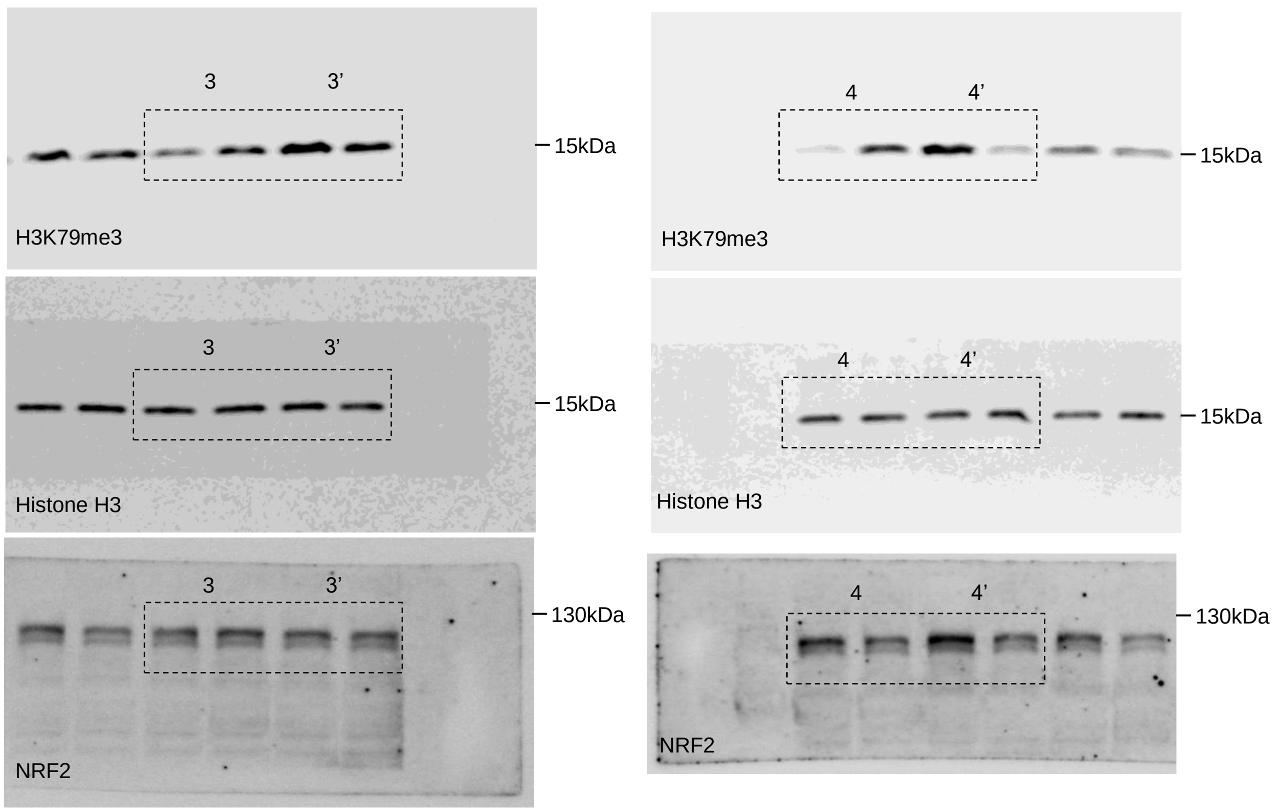

Figure 7d

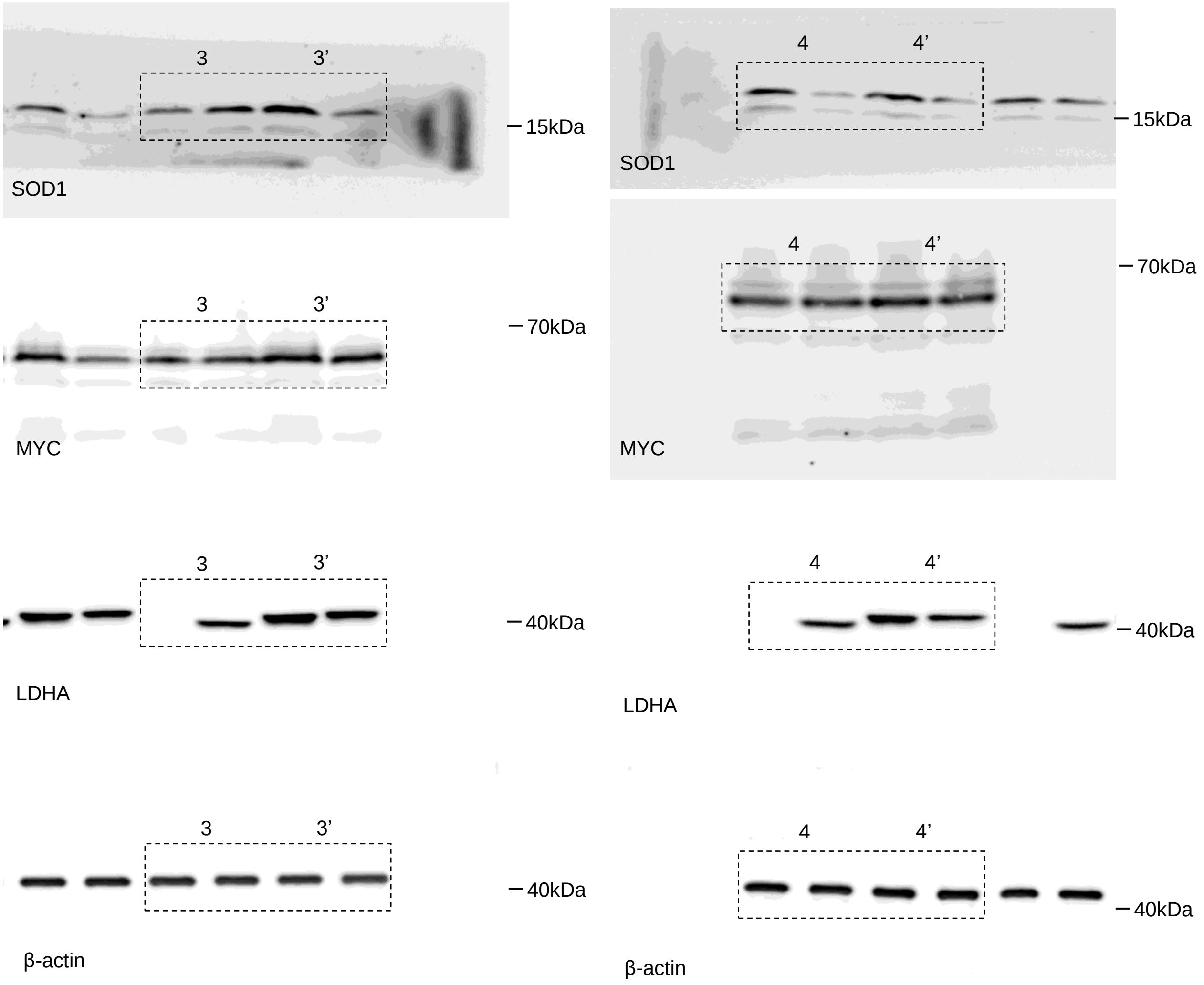

Supplementary Figure 2a

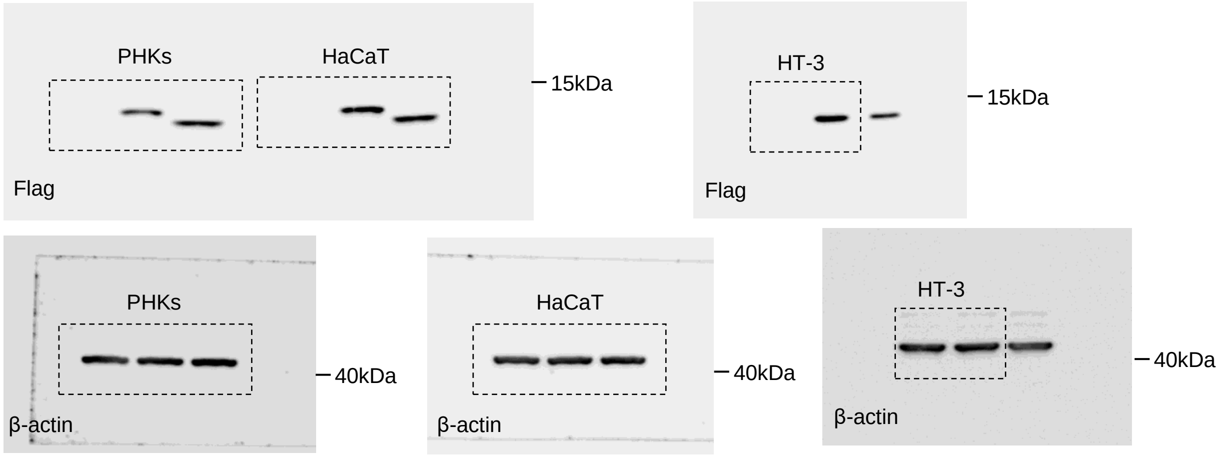

Supplementary Figure 6f

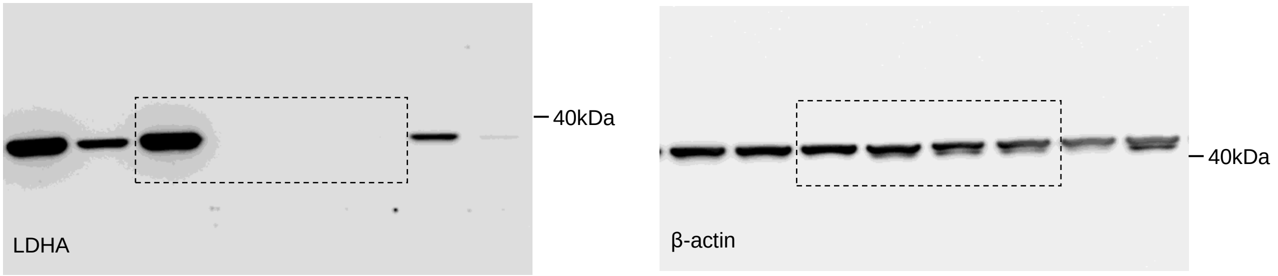

Supplementary Figure 7b

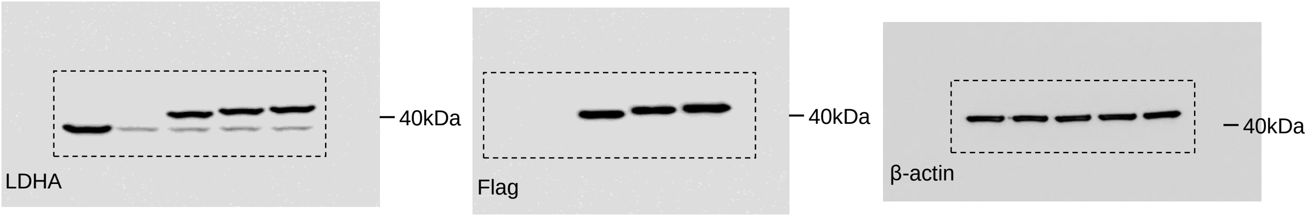

Supplementary Figure 8a

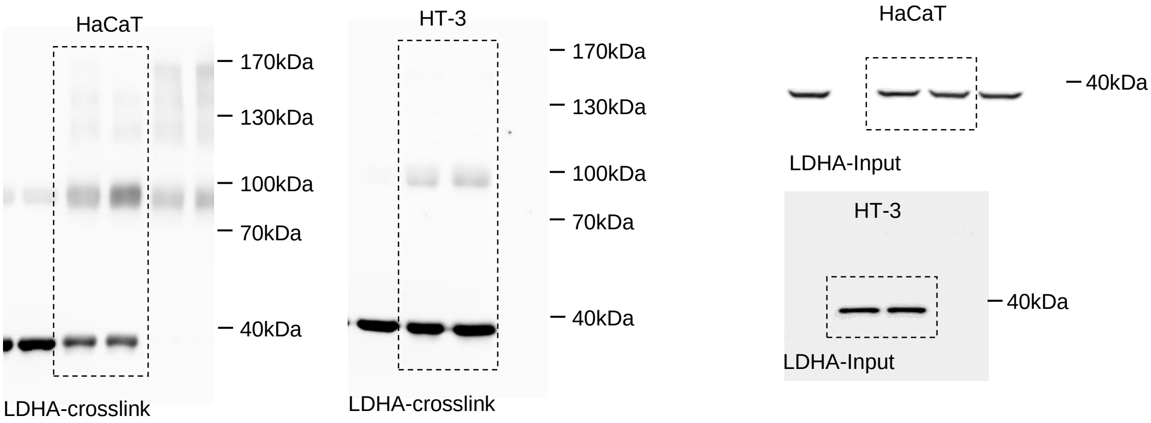

Supplementary Figure 8d

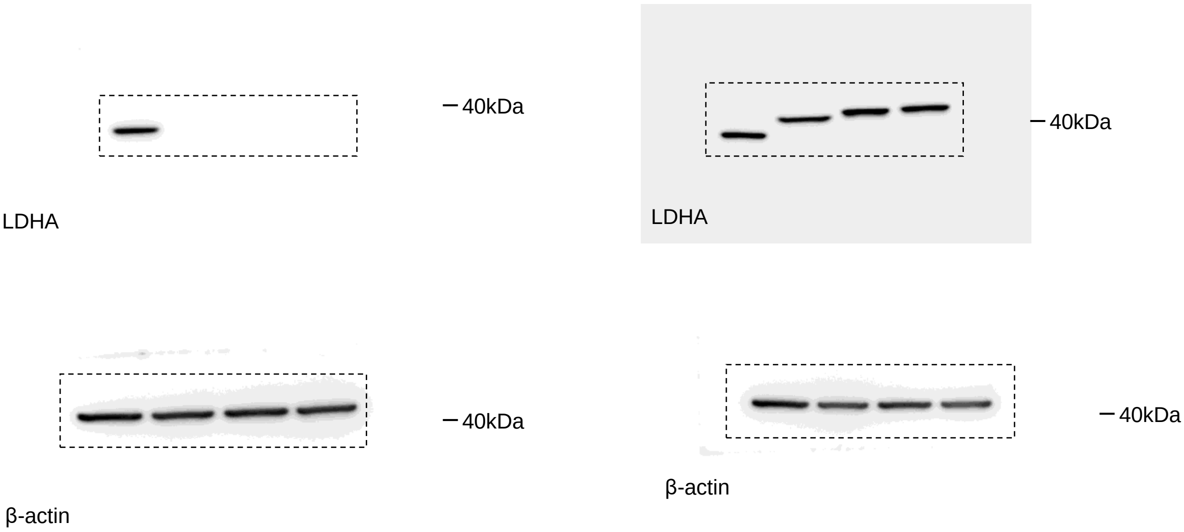

Supplementary Figure 9a

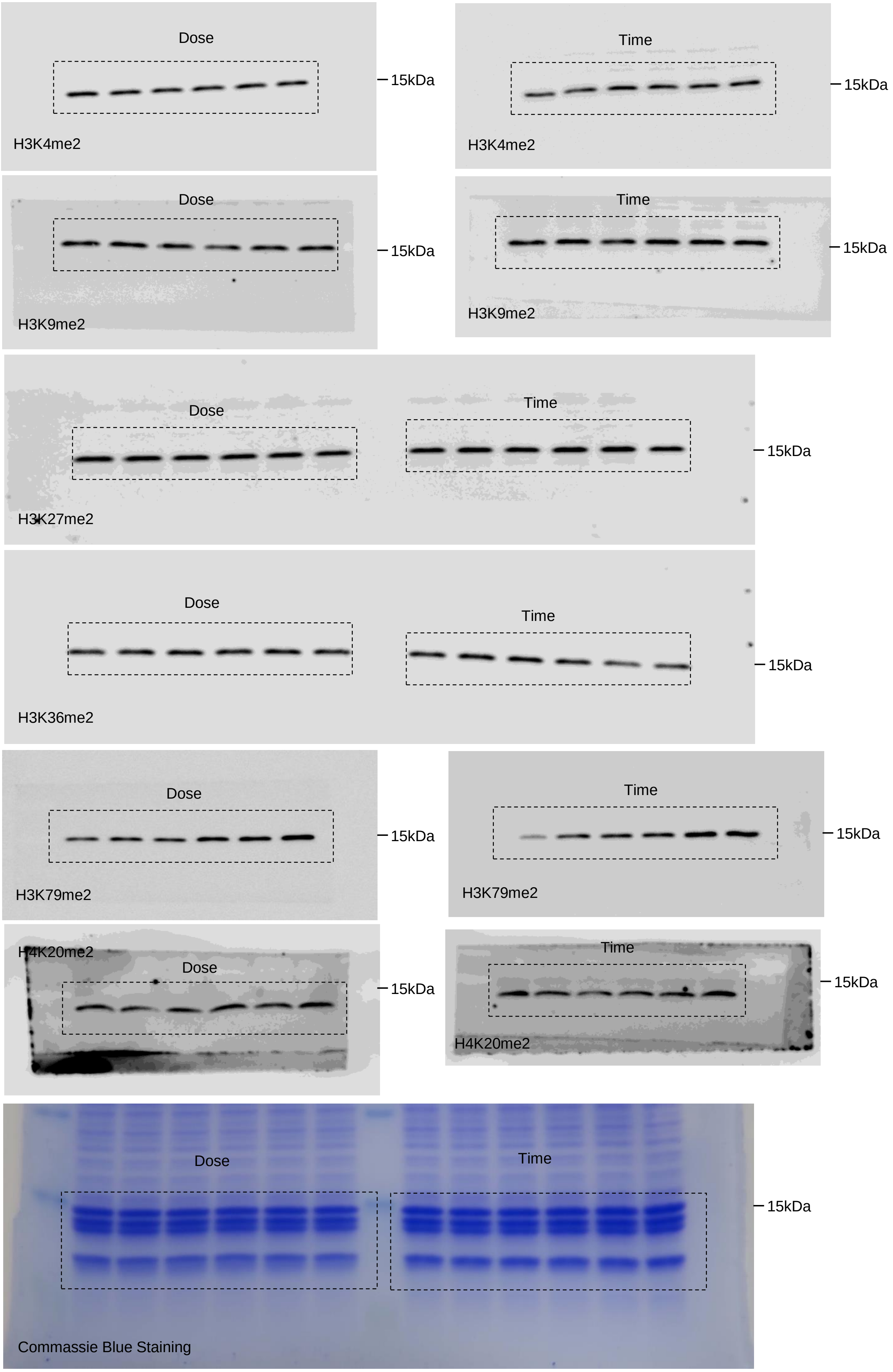

Supplementary Figure 9b

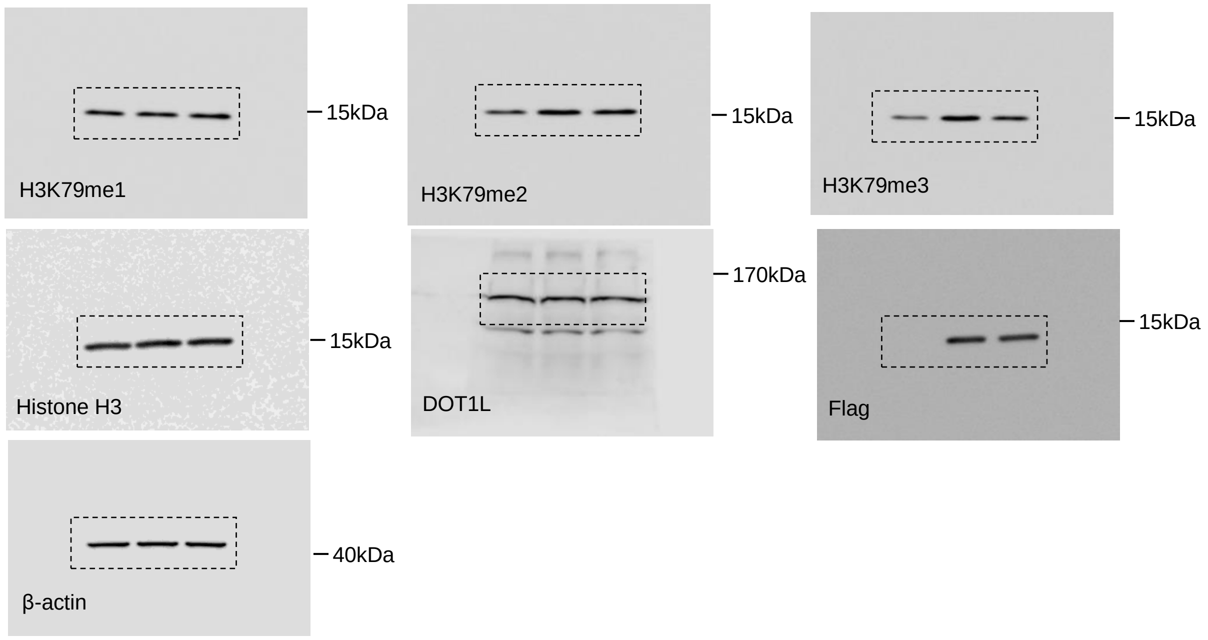

Supplementary Figure 9c

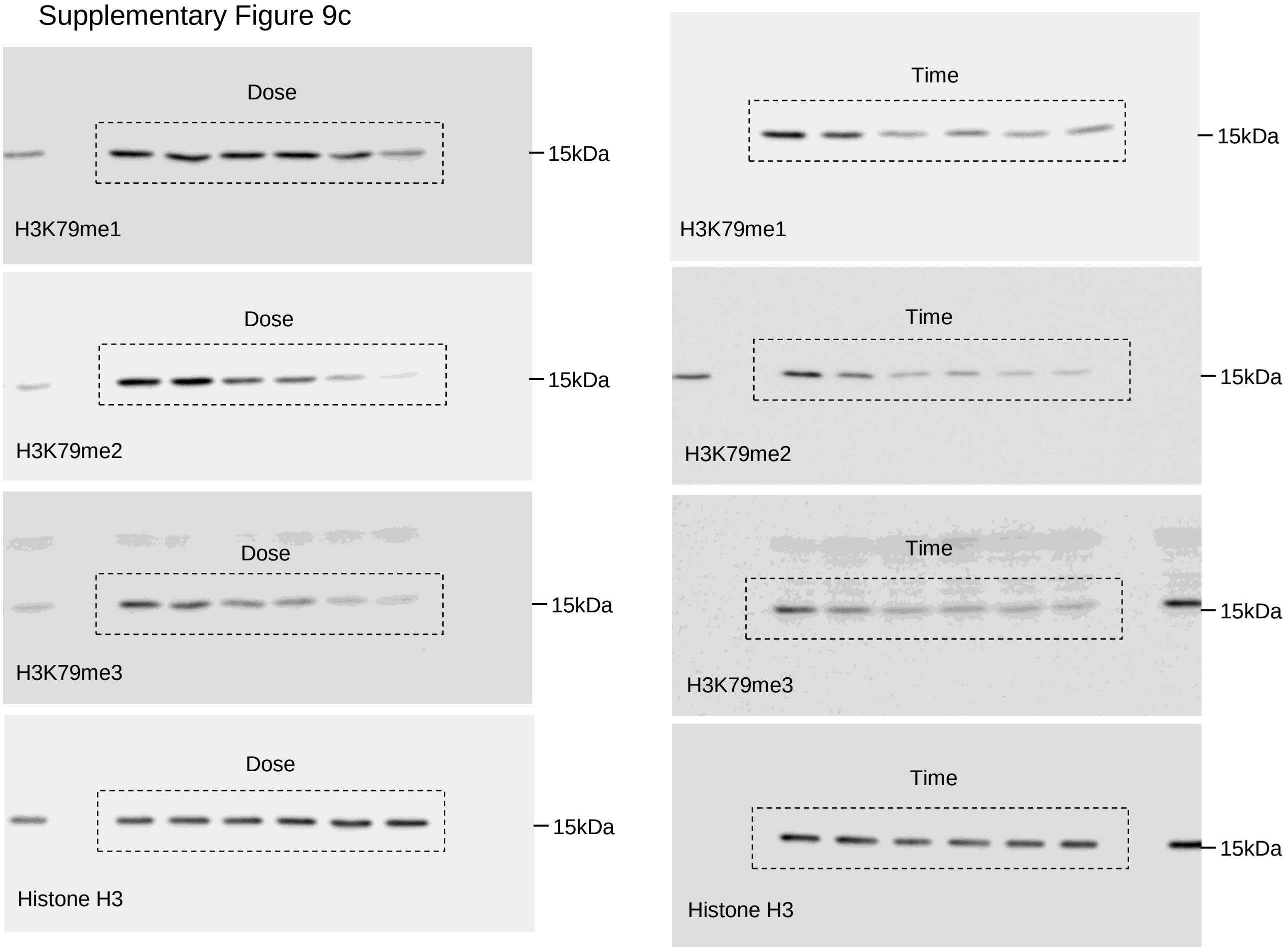

Supplementary Figure 9d

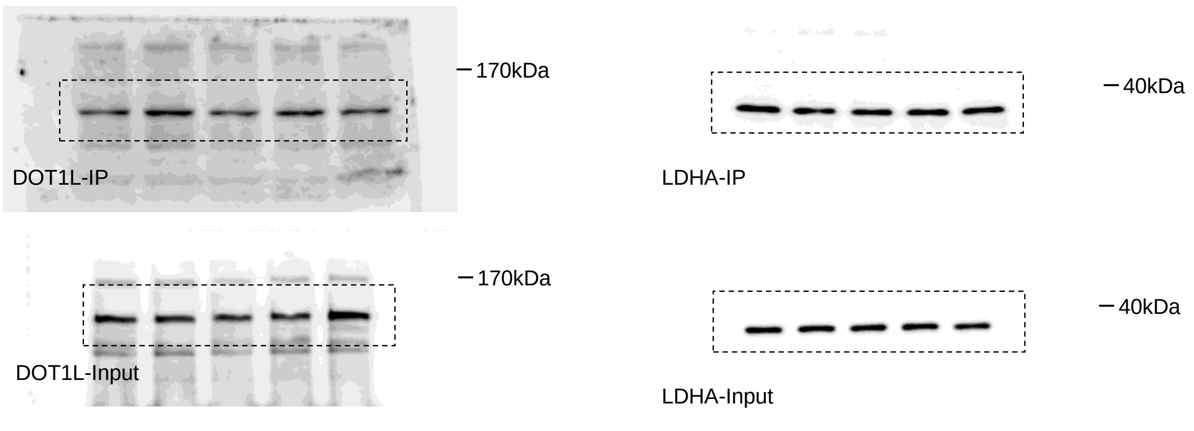

Supplementary Figure 11b

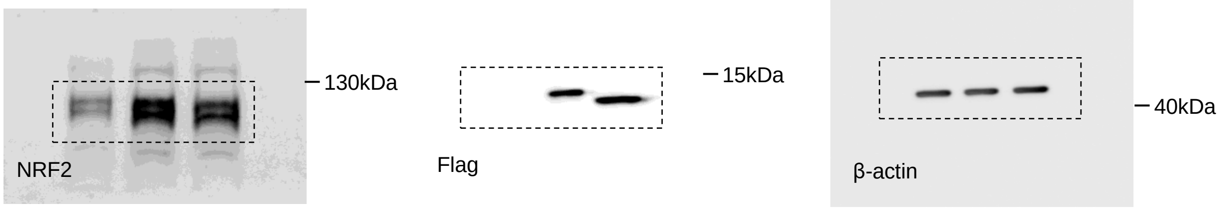

Supplementary Figure 11c

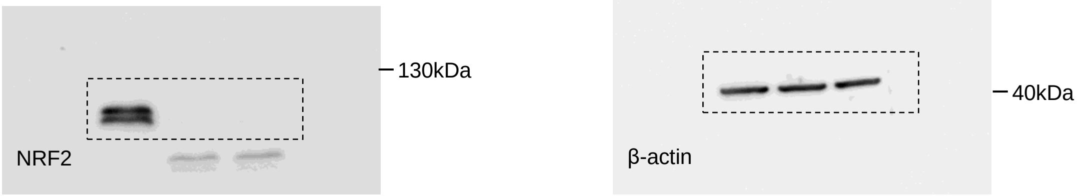

Supplementary Figure 11f

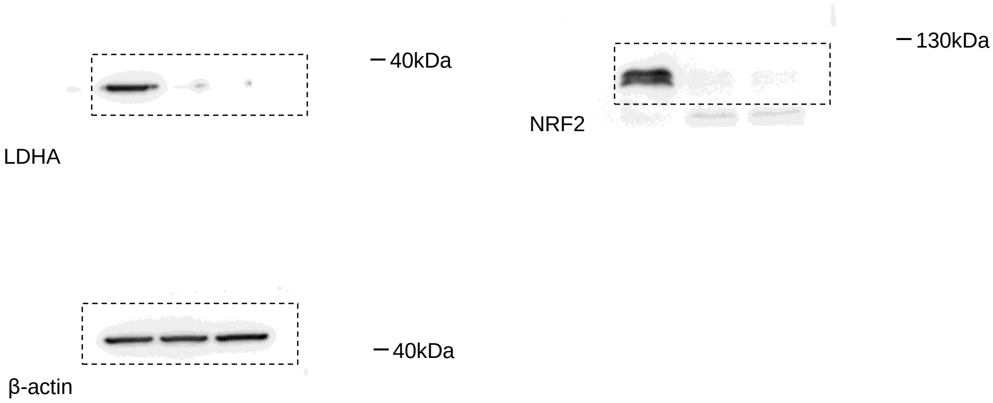

Supplement: Supplementary file 1 — Supplementary Information [file 41467_2018_6841_MOESM1_ESM.pdf]
