# Supplementary material for: Nuclear lactate dehydrogenase A senses ROS to produce α-hydroxybutyrate for HPV-induced cervical tumor growth
Source: Nat Commun. 2018 Oct 24;9:4429. doi: 10.1038/s41467-018-06841-7 (PMC6200739; doi:10.1038/s41467-018-06841-7)
Supplement: Supplementary file 3 — Description of Additional Supplementary Files [file 41467_2018_6841_MOESM3_ESM.docx]

**Description of Additional Supplementary Files**

File Name: Supplementary Data 1

Description: Potential redox sensors responding to HPV infection.

File Name: Supplementary Data 2

Description: HR HPV-type characterization of 66 cervical specimens.

File Name: Supplementary Data 3

Description: Quantitative RT-PCR primer sequences.
